# Silk Fibroin for Biomedical Applications with Emphasis on Bioimaging, Biosensing and Regenerative Systems: A Review

**DOI:** 10.3390/molecules31071142

**Published:** 2026-03-30

**Authors:** Snjezana Tomljenovic-Hanic, Asma Khalid

**Affiliations:** School of Physics, University of Melbourne, Parkville, VIC 3010, Australia; snjezana.thanic@unimelb.edu.au

**Keywords:** silk fibroin, regenerated silk, bioimaging and biosensing, wound healing, tissue engineering, drug delivery, optical fibre sensors, wearable and edible sensors

## Abstract

Biomaterials are engineered to interact with biological systems for therapeutic or diagnostic purposes. Among them, natural biomaterials offer important advantages over many synthetic polymers, including intrinsic biocompatibility, non-toxicity and biodegradability. Silk fibroin, a fibrous protein derived mainly from *Bombyx mori* cocoons, has re-emerged as a particularly versatile platform because it combines favourable mechanical, thermal, electrical and optical properties with aqueous processing and tuneable degradation. In this review, we first summarise the key structural, physicochemical and functional properties of regenerated silk fibroin, including its mechanical behaviour, thermal stability, dielectric and piezoelectric response, optical transparency and low autofluorescence. We then describe how extraction and regeneration protocols are used to produce defined material formats—fibres and nanofibrous mats, porous 3D scaffolds and hydrogels, sub-micron particles, thin films and microstructured devices—and outline major functionalisation strategies, ranging from physical blending and encapsulation to covalent chemistry, genetic engineering of recombinant silk variants, and enzyme-mediated conjugation approaches. Building on this foundation, we critically examine biomedical applications of silk fibroin with a particular emphasis on (i) hybrid silk–fluorophore systems for bioimaging and biosensing (nanodiamonds, quantum dots and organic dyes), (ii) optical fibre, wearable and edible sensors for health and food monitoring, (iii) wound dressings and wound-sensing platforms, and (iv) tissue engineering scaffolds and drug-delivery depots. Finally, we discuss current limitations, including process variability, the trade-offs introduced by blending and cross-linking, and the challenges posed by non-degradable inorganic fillers and clinical translation. Together, these perspectives highlight silk fibroin’s potential and constraints as a multifunctional biomaterial for next-generation biomedical devices and theranostic systems.

## 1. Introduction

Biomaterials are defined as synthetic or natural materials that interact with biological systems. Biomaterials are utilised for a wide spectrum of medical applications. Those include bioimaging, biosensing, drug delivery, tissue engineering, implants, and medical devices. Various materials including metals, polymers, ceramics, etc., were explored and applied as biomaterials, but natural biomaterials that are extracted from living organisms have many advantages over synthetic biomaterials, such as non-toxicity, biocompatibility, and biodegradability [1]. In particular, biodegradability is advantageous for many short-term biomedical applications, such as bioimaging and drug delivery. However, biodegradability can be a disadvantage in some applications where the stability and longevity of medical products are essential, and those requirements apply for body implants. Importantly, for some natural biomaterials, biodegradability can be engineered, as is the case for silk fibroin [2].

Silk fibroin (SF) is a biocompatible natural protein polymer, primarily derived from *Bombyx mori* silkworms. There are other sources of SF, such as flies, spiders, mites, and scorpions that synthesise SF. Despite the different sources and consequent protein structures, these silks have typically high levels of protein crystallinity and similar amino acidic compositions [2]. However, silkworms are the most abundant, inexpensive, and accessible natural source, mainly due to the long history of domesticating silkworms for silk production. SF is degradable and exhibits a low inflammatory response in vivo [3]. SF has emerged as a biomaterial for numerous biomedical applications due to its unique combination of biocompatibility, mechanical robustness, tuneable biodegradability and excellent optical properties [4,5]. SF is a material that degrades at a controlled rate in vivo and in vitro [6]. The final breakdown products of SF degradation are the corresponding amino acids, which are easily absorbed in vivo [7]. This is one of the reasons for the extensive use of SF in the field of biomedicine [3]. The degradability of SF can be engineered from weeks to years by modifying the crystallinity and β sheet content, simply by processing the fibroin or using post-processing methods [8].

Silk fibroin from *Bombyx mori* consists primarily of a heavy chain (~390 kDa) and a light chain (~26 kDa) linked via disulfide bonds, associated with a glycoprotein P25 in a 6:6:1 molar ratio [9]. The heavy chain contains highly repetitive glycine- and alanine-rich domains, particularly the glycine–alanine–glycine–alanine–glycine–serine GAGAGS motif, which promotes the formation of antiparallel β-sheet crystallites (Silk II structure). These nanocrystalline β-sheet domains are interspersed with less ordered amorphous regions that contain more bulky and polar amino acids. This hierarchical organisation—β-sheet nanocrystals embedded within a semi-amorphous protein matrix—governs the mechanical and degradation behaviour of regenerated silk fibroin. The β-sheet domains provide stiffness and tensile strength, whereas amorphous regions contribute elasticity and facilitate hydration-dependent degradation. Structural transitions between Silk I (random coil/α-helix) and Silk II (β-sheet) can be induced through solvent treatment, shear, pH change, or thermal processing, enabling the programmable control over crystallinity and material performance [9,10]. This structure–property coupling is central to understanding silk fibroin’s mechanical robustness, tuneable biodegradability, optical transparency, and compatibility with functionalization strategies discussed throughout this review.

Even though silk is one of the lightest structural materials, its tensile strength is superior to many commonly used biomaterials [4]. SF’s mechanical properties—such as its high tensile strength, toughness and elasticity—can be tuned to closely mimic those of soft and hard tissues, thereby reducing the risk of mechanical mismatch, inflammation or damage when implanted [4]. The formation and extent of β-sheet (Silk II) crystallites within the protein network largely determine the stiffness, toughness and degradation rate [11]. Recent reviews have summarised strategies to modulate β-sheet content and hence mechanics, including chemical, physical and enzymatic crosslinking, water and alcoholic treatments, irradiation, blending with other polymers or inorganic fillers, and a careful control of concentration, temperature, humidity and pH during processing [12,13].

The thermal behaviour of SF is also favourable for many biomedical and device applications. The water content within the material and its crystallinity determine the characteristic thermal transitions, including glass transition and the onset of thermal denaturation [14]. Across different processing routes (films, fibres, sponges and particles), SF materials consistently exhibit a good thermal stability, with no clear thermal degradation before ~200 °C under dry conditions [15,16]. The processing history (e.g., solvent system, annealing, β-sheet content) shifts these transitions slightly, but, overall, SF maintains structural integrity over the range of temperatures relevant for sterilisation, storage and standard biomedical handling [11]. Electrically, SF behaves as an insulating protein in the dry state, with very low conductivity and dielectric losses [14]. Upon hydration, however, proton and ion transport through the amorphous domains leads to a marked increase in ionic conductivity, which can be further tuned by controlling the water content and salt concentration or by forming composites with conductive fillers [4]. This humidity-dependent electrical response has been exploited in emerging silk-based flexible electronics and sensors, where SF serves as a biocompatible dielectric, ion-conducting matrix or supporting substrate for active electronic components [4].

Furthermore, SF possesses excellent optical properties that are highly advantageous for photonic, imaging and sensing applications. SF is more than 90% transparent in the visible region and remains highly transmissive into the near-infrared, with very low intrinsic autofluorescence and scattering compared with many synthetic polymers or collagen-based substrates [17,18]. The refractive index of SF (typically ~1.54–1.56 in the visible) can be adjusted with the processing conditions and regeneration protocols, enabling a control over light confinement and guiding in silk-based optical components [19]. Notably, native gland fibroin exhibits a slightly higher refractive index (*n* ≈ 1.566 at 500 nm) and absorption coefficient than regenerated SF, highlighting how processing routes impact optical performance. These combined features have supported the fabrication of a broad range of SF photonic structures, including planar films, optical fibres, printed and planar waveguides, diffraction gratings, and microspheres [20,21,22,23,24].

Although the majority of biomedical studies employ SF derived from *Bombyx mori* cocoons, fibroin proteins obtained from wild silks such as *Antheraea pernyi* (Tussah silk) exhibit distinct molecular features that may influence biological performance and processing behaviour [25]. The heavy-chain fibroin of *Bombyx mori* is characterised by highly repetitive GAGAGS motifs that promote dense β-sheet nanocrystal formation and confer a high tensile strength and structural stability. However, *Bombyx mori* fibroin lacks intrinsic cell-adhesive peptide motifs such as arginine–glycine–aspartic acid (RGD), which are often required to enhance integrin-mediated cell attachment [25]. As a result, *Bombyx mori*-based scaffolds are frequently functionalized through blending, peptide conjugation, or recombinant modification to improve cell–material interactions.

In contrast, fibroin derived from *Antheraea pernyi* contains naturally occurring RGD-like sequences within its protein backbone, which can promote enhanced cell adhesion without additional surface modification. Comparative studies evaluating *Antheraea* and *Bombyx mori* SF have reported the improved attachment and spreading of fibroblast cells on *Antheraea*-derived matrices, attributed to the presence of integrin-binding motifs and differences in protein composition and structure [26,27]. These findings suggest that intrinsic sequence-level differences between silk species can influence cell–material interactions without requiring post-processing functionalization.

Beyond biochemical motifs, structural and processing differences are also observed. *Antheraea* silk typically exhibits a higher intrinsic crystallinity and different amino acid distributions, which can influence solubility, dissolution behaviour, and regeneration protocols. Compared with *Bombyx mori* silk, *Antheraea* fibroin may require modified degumming or solvent systems to achieve reproducible aqueous regeneration. Variations in the molecular weight distribution after dissolution can further affect the mechanical properties and degradation kinetics of regenerated scaffolds [25].

From a translational perspective, *Bombyx mori* remains the dominant source for biomedical applications due to its established sericulture infrastructure, reproducibility, and regulatory familiarity [28]. However, *Antheraea*-derived fibroins offer intriguing advantages for applications where enhanced intrinsic cell adhesion or specific mechanical profiles are desired [26,27]. Comparative and hybrid approaches may therefore provide opportunities to tailor bioactivity and mechanics at the protein sequence level, rather than relying solely on post-processing functionalization. These source-dependent differences highlight that silk fibroin is not a chemically uniform material but rather a family of related structural proteins whose molecular architecture directly influences biological performance, processing parameters, and application-specific suitability. Collectively, the mechanical, thermal, electrical, and optical characteristics discussed above underpin SF’s versatility as a platform for multifunctional biomedical devices.

**Figure 1:**
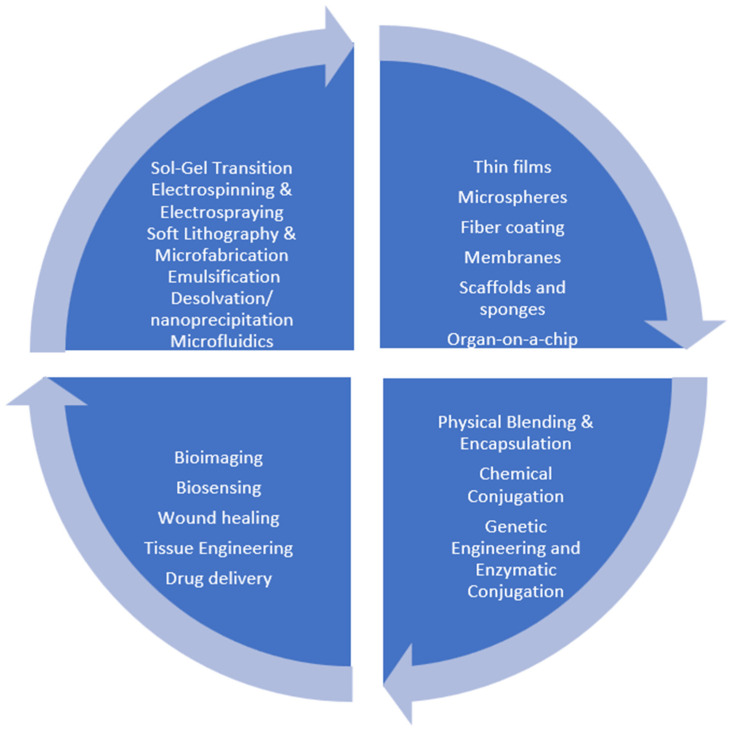
Schematic of this review content including fabrication, SF structures considered, functionalization and biomedical applications.

This review aims to provide a chemically grounded overview of regenerated silk fibroin as a multifunctional biomedical material, with a particular emphasis on structure–property relationships that enable optical, sensing, regenerative, and drug-delivery applications. While numerous reviews have addressed individual aspects of silk fibroin, here, we integrate molecular structure, processing strategies, functionalization chemistry, and representative biomedical platforms within a unified framework. Attention is given to imaging- and biosensing-enabled systems, while broader regenerative and therapeutic applications are discussed to contextualise translational potential. Figure 1 schematically summarises the logical flow of the manuscript, linking molecular structure and fabrication methods to material formats, functionalization strategies, and representative biomedical applications. The schematic highlights the logical progression from protein composition and β-sheet structure to fabrication strategies and finally to imaging, sensing, regenerative and therapeutic systems. By visually linking structure, processing and application domains, this figure clarifies the integrated framework adopted throughout the manuscript.

## 2. Fabrication and Functionalization

The novel intrinsic properties of SF are only fully realised through advanced fabrication techniques that transform the raw protein into tailored architectures. The process begins with the extraction and purification of SF from the cocoons of the *Bombyx mori* silkworm, a critical step that determines the quality and consistency of the final material. This is typically achieved through a well-established protocol [10] of degumming—boiling the cocoons in an alkaline solution, most commonly sodium carbonate (Na_2_CO_3_), to remove the sericin glue protein—followed by dissolving the purified fibroin fibres in a high-concentration salt solution, such as lithium bromide (LiBr). While alkaline degumming with Na_2_CO_3_ effectively removes sericin, excessive treatment can reduce fibroin molecular weight through chain scission, thereby influencing the mechanical strength and degradation behaviour of regenerated materials [29]. Similarly, dissolution in concentrated LiBr (typically 9.3 M) enables efficient fibroin solubilization but may alter molecular weight distribution depending on the temperature and duration of exposure [29]. Variations in molecular weight directly affect the viscosity, gelation kinetics, and final β-sheet content after regeneration. Alternative solvent systems, such as CaCl_2_/ethanol/water, have also been explored to modulate dissolution efficiency and structural preservation [10]. Therefore, extraction and regeneration protocols represent critical upstream determinants of downstream biomedical performance. The resulting aqueous SF solution obtained after degumming and dissolving in LiBr is then dialyzed against water to remove salts, yielding a regenerated SF solution that serves as the versatile “building block” for all subsequent fabrication. The versatility of SF is demonstrated by the wide array of processing techniques used to fabricate structures across multiple length scales, from bulk materials to micro- and nano-architectures, each suited for specific biomedical applications. These methods can be broadly categorised based on the final material format [10].

As summarised in Table 1, these processing routes are used to produce fibres and fibrous mats, 3D porous scaffolds and sponges, bulk hydrogels, particulate systems, thin films and coatings, and microstructured devices; in the following subsections, we describe the main fabrication principles for each class.

### 2.1. Sol–Gel Processing and Cryogelation (Hydrogels and Porous Sponges)

One of the most straightforward methods to form solid SF structures is through the induction of a sol–gel transition. Aqueous SF solutions are metastable and can be triggered to undergo a conformational change from a random coil/alpha-helix (Silk I) to a β-sheet (Silk II) structure, leading to physical crosslinking and gelation. This transition can be initiated by various stimuli, including sonication [2], vortexing, changes in pH or ionic strength, and exposure to polar solvents (e.g., methanol or ethanol) or heat [12]. The kinetics of gelation and the final mechanical properties of hydrogel are highly tuneable based on the SF concentration, temperature, and gelation method employed.

For applications requiring highly porous scaffolds, such as tissue engineering, cryogelation is a powerful technique [30]. This process involves freezing the SF solution, which leads to the formation of ice crystals that act as a porogen. The SF is concentrated in the walls between the growing ice crystals. The subsequent sublimation of the ice via lyophilization results in a macroporous sponge-like scaffold with interconnected pores that facilitate cell infiltration and nutrient diffusion.

### 2.2. Electrospinning and Electrospraying (Fibres and Fibrous Mats)

Electrospinning is one of the most widely researched techniques for fabricating non-woven mats of micro- and nanofibres that closely mimic the topography and scale of the native extracellular matrix (ECM). In this process, a high voltage is applied to a droplet of SF solution, creating a charged jet that is accelerated toward a grounded collector. As the jet travels, the solvent evaporates, leaving behind solid, continuous fibres. By carefully controlling parameters such as the SF concentration, viscosity, applied voltage, and collector type, researchers can tailor fibre diameter, alignment, and mat porosity [13]. These nanofibrous mats exhibit a high surface-area-to-volume ratio, making them excellent for wound dressings, tissue scaffolds, and sensing platforms. A related technique, electrospraying, uses similar principles but with lower polymer concentrations or different flow rates to produce particles instead of fibres. This method is effective for generating SF micro- and nanoparticles and fibres for imaging, sensing and drug release applications in a single step [31].

### 2.3. Photolithography and Soft Lithography for Microstructured Silk Devices

**Figure 2:**
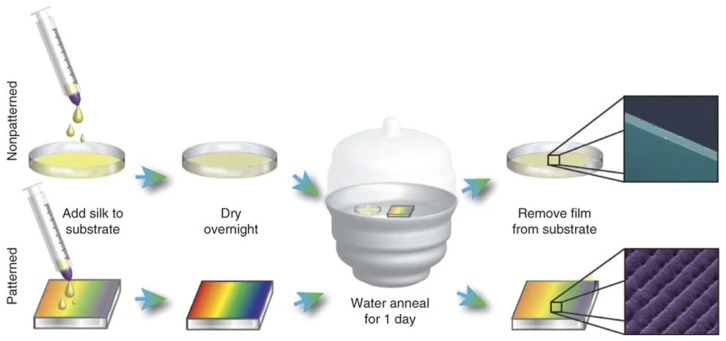
Representative material formats of regenerated silk fibroin obtained via aqueous processing routes. The figure illustrates the transformation of regenerated silk fibroin solution into optically transparent thin films and patterned coatings using casting and spin-coating approaches, as well as micro- and nano-fabrication strategies for generating functional architectures. These planar and microstructured silk formats provide mechanically stable, biocompatible substrates for subsequent functionalization and integration into biomedical devices. Reproduced from [10] under open access terms.

For advanced biomedical applications like organ-on-a-chip models, implantable biosensors, and structured tissue scaffolds, SF can be fabricated into precise microscale patterns using techniques adapted from the semiconductor industry. The ability to pattern SF at this level allows for an unprecedented control over cell environments and device functionality. A significant innovation is the development of a photosensitive silk “resist” [32]. By chemically modifying SF to make it reactive to light, researchers can use a technique called photolithography. In this process, a thin film of this photosensitive silk is exposed to patterned ultraviolet (UV) light. The light hardens the exposed areas, making them water-insoluble, while the unexposed regions remain soluble and are washed away during development. This effectively “prints” a high-resolution design onto the silk layer, enabling the creation of intricate structures like microfluidic channels, optical elements, and electrode patterns entirely from biocompatible silk [32,33]. Alongside photolithography, soft lithography techniques like replica moulding are widely used for their simplicity and effectiveness [34]. This involves casting an aqueous SF solution onto a pre-patterned mould. After drying and solidifying, the silk film is peeled away, retaining the inverse pattern of the mould. This method is exceptionally valuable in tissue engineering for creating surfaces with specific micro-grooves or ridges. These patterned surfaces can contact-guide cells, promoting aligned growth, which is crucial for regenerating anisotropic tissues like nerves, cardiac muscle, and tendons [34]. Together, these microfabrication methods elevate SF from a bulk material to a precision-engineered platform. They enable the creation of all-silk devices that integrate structural, optical, and biological functions into a single, biocompatible, and resorbable system for next-generation medical technologies. Examples of patterned silk fibroin films produced using these approaches are illustrated in Figure 2.

### 2.4. Fabrication of Nanoparticles and Microspheres (Particulate Systems)

SF particulate systems, including nanoparticles and microspheres, have been extensively investigated as carriers for controlled and targeted drug delivery due to their biocompatibility, biodegradability, and tuneable physicochemical properties. In these systems, the fabrication route plays a decisive role in determining particle size, size distribution, morphology, drug encapsulation efficiency, and release kinetics, all of which directly influence biological performance and therapeutic efficacy [35].

SF nanoparticles typically refer to particles in the sub-100 nm to ~200 nm size range and are most commonly produced using desolvation or nanoprecipitation approaches, in which a water-miscible non-solvent (such as ethanol or acetone) induces controlled protein aggregation [24]. These nanoscale carriers are particularly suited for intracellular drug delivery, enhanced cellular uptake, and systemic administration, owing to their small size and large surface-to-volume ratio. By adjusting processing parameters such as the silk concentration, solvent composition, and post-treatment conditions, nanoparticle stability and degradation behaviour can be finely tuned to achieve sustained or stimulus-responsive release profiles [10].

In contrast, SF microspheres and sub-micron spheres are defined primarily by their spherical geometry and are typically fabricated using emulsification techniques (e.g., water-in-oil emulsions) or microfluidic co-flow methods. These approaches enable the production of spherical carriers with well-defined size distributions and higher loading capacities, making them particularly attractive for depot-type drug delivery, imaging, and combined theranostic applications. Microfluidic strategies, in particular, allow a precise control over the sphere diameter, β-sheet content, and degradation rate, enabling the reproducible fabrication of monodisperse carriers with predictable release behaviour [36]. Together, these particulate fabrication strategies highlight the versatility of SF as a platform material for nanoscale and microscale delivery systems, where both particle size and geometry can be tailored to meet specific biomedical requirements, ranging from intracellular delivery to long-term controlled release depots [10,35,36].

#### 2.4.1. Emulsification

This is a standard top-down method for fabricating SF microspheres, typically using a water-in-oil (W/O) process. An aqueous SF solution is homogenised into a continuous oil phase containing a surfactant, where parameters such as the shear force, surfactant concentration, and viscosity ratio of the phases dictate the final particle size distribution [37,38]. The liquid SF droplets are subsequently solidified to form stable particles. This is primarily achieved through chemical crosslinking, often by adding glutaraldehyde to the oil phase to create covalent bonds between SF chains. Alternatively, physical solidification can be induced by exposing the emulsion to methanol vapour or heat, which promotes β-sheet formation and renders the particles insoluble. The choice of solidification mechanism directly impacts the degradation rate and drug release profile of the resulting microparticles. A key advantage of this method is its high encapsulation efficiency for hydrophilic therapeutic agents, which are dissolved in the aqueous SF phase prior to emulsification and become entrapped within the particle matrix during crosslinking [37,38].

#### 2.4.2. Desolvation/Nanoprecipitation

This is a bottom-up technique ideal for producing smaller, sub-200 nm nanoparticles. This method relies on a controlled reduction in SF’s solubility in water. A desolvating agent, which is a miscible non-solvent for SF (such as ethanol, acetone, or potassium phosphate), is added dropwise to an agitated aqueous SF solution. As the non-solvent ratio increases, the SF chains dehydrate and undergo a conformational change, self-assembling into homogeneous nanoparticles [39]. The process is delicate; the rate of addition, final non-solvent concentration, and ionic strength of the solution all critically influence particle size and polydispersity. The initially formed nanoparticles are often metastable and require stabilisation via crosslinking with agents like genipin. This method offers an excellent control over size and is particularly suited for encapsulating hydrophobic drugs, which can be pre-dissolved in the non-solvent phase before its addition [40]. The choice of fabrication method directly impacts the drug release profile. Particles made via a high β-sheet content (e.g., through methanol treatment) tend to degrade more slowly, providing sustained release, while those with less crystalline structures release their payload more rapidly. This tuneability, combined with the ability to functionalise the SF surface with targeting ligands, makes SF particulate systems a highly versatile platform for precision medicine.

#### 2.4.3. Microfluidic Fabrication

This offers a highly controlled method for producing uniform SF particles. This approach, as detailed in the referenced works, utilises microfluidic devices to generate monodisperse droplets of an aqueous SF solution within a continuous oil phase [24]. The precise manipulation of fluid flow rates within micron-scale channels allows for an exceptional control over the droplet size and, consequently, the final particle diameter. A critical advancement of this technique is the in situ solidification process. The organic solvent present in the carrier oil phase diffuses across the oil–water interface into the aqueous silk droplet. This solvent exchange initiates a dehydration of the SF, prompting a rapid conformational transition from a random coil to a β-sheet structure. This process solidifies the droplet into a stable protein particle without the requirement for chemical crosslinkers, preserving the innate biocompatibility of silk [24,41,42]. The result is a continuous and scalable production of smooth, monodisperse particles with precisely tuneable sizes and defined crystalline content, which is paramount for achieving consistent and predictable drug loading and release kinetics in therapeutic applications.

### 2.5. Casting and Spin-Coating (For Thin Films and Surface Coatings)

The film-forming capability of SF is central to its use in creating robust, ultra-thin, and optically transparent coatings for a diverse range of biomedical interfaces. These coatings are primarily fabricated using techniques such as spin-coating or drop-casting, where an aqueous SF solution is applied to a substrate and allowed to dry under controlled conditions. As illustrated in Figure 2, regenerated silk fibroin solutions can be transformed into optically transparent planar films and patterned coatings through casting and spin-coating methods. The referenced study [10] demonstrates the formation of uniform thin films with a controlled thickness, as well as microstructured architectures generated through subsequent lithographic or templating strategies. The images highlight both the optical clarity and structural fidelity of silk films following β-sheet induction, confirming their mechanical stability and suitability as functional substrates. These planar silk formats serve as foundational platforms for further chemical functionalization, biosensing interfaces, optical waveguides and implantable coatings, thereby linking fundamental aqueous processing to advanced biomedical device integration.

A critical post-processing step for these films, such as treatment with methanol or water vapour annealing, is required to induce a conformational transition to a β-sheet structure, rendering the coatings water-insoluble and mechanically stable while directly tuning their degradation rate [10]. The utility of SF coatings stems from their multifunctional nature. They serve as a biocompatible interface, significantly improving the integration of implants and reducing foreign body responses. A key advancement is the creation of hybrid materials, where SF is used to coat other nanomaterials to enhance their biocompatibility and functionality. For instance, coating nanodiamond (ND) particles with SF creates a stable, biocompatible hybrid material (ND-SF) that mitigates nanodiamond aggregation and improves its dispersion in biological environments, enhancing its potential for drug delivery and bioimaging [18].

Furthermore, SF coatings are highly transparent across a broad wavelength range and exhibit a refractive index that is well matched to biological tissues. This makes them particularly valuable for optical sensing applications. As demonstrated, a thin SF coating can be effectively applied to an optical fibre core. This bio-derived coating not only protects the fibre but also acts as a functional biorecognition layer. Its high transparency ensures minimal optical loss, while its ability to immobilise biomolecules enables the development of highly sensitive biosensors for detecting various analytes [43]. The selection of fabrication parameters provides precise control over the coating’s thickness, morphology, and functional performance, making it a powerful tool for advanced surface engineering in medicine and sensing. Examples of regenerated silk fibroin films fabricated via casting, spin-coating, and lithographic patterning are shown in Figure 2. These formats exemplify how processing parameters influence thickness, surface morphology, and structural organisation, which in turn determine optical and mechanical performance, as discussed in Section 2.5.

### 2.6. Functionalization of Silk Fibroin

The expansion of SF into advanced biomedical roles is critically dependent on strategies to functionalise the material, enhancing its native properties with tailored bioactivity, sensing capability, and therapeutic function. These methods are broadly classified into physical blending, chemical conjugation, and biological modification, each offering distinct advantages for specific applications.

#### 2.6.1. Physical Blending and Encapsulation

This is the most straightforward functionalization technique, involving the direct mixture of active compounds with an aqueous SF solution prior to processing into the final material format (e.g., films, fibres, hydrogels) [13]. The mechanism relies on physical entrapment within the SF matrix and secondary interactions such as hydrogen bonding, hydrophobic interactions, and electrostatic forces. This method is highly effective for loading a wide range of cargo, including small molecule drugs (e.g., chemotherapeutics like doxorubicin [44]), antibiotics, and nanoparticles. For instance, the blending of fluorescent nanodiamonds into an SF solution prior to electrospinning creates composite membranes that leverage the diamond’s stable fluorescence for biosensing and the silk’s biocompatibility for wound healing [31]. A key limitation is the potential for burst release, as the cargo is not covalently tethered, making release kinetics highly dependent on the diffusion rate and the degradation profile of the SF matrix.

#### 2.6.2. Chemical Conjugation

This approach forms stable, covalent bonds between functional molecules and reactive amino acid side chains on the SF protein. This provides a precise, sustained presentation of bioactive motifs and prevents the premature leaching of cargo. Common targets for conjugation include the primary amines of lysine residues, carboxylic acids of aspartic and glutamic acids, and hydroxyl groups of tyrosine [45,46].

The use of EDC (1-ethyl-3-(3-dimethylaminopropyl) carbodiimide) with NHS (N-hydroxysuccinimide) is a commonly used technique for covalently conjugating biomolecules to SF. This reaction targets carboxylic acid (-COOH) groups on aspartic acid, glutamic acid, or the C-terminus of SF, activating them with EDC to form an unstable O-acylisourea intermediate. The addition of NHS stabilises this intermediate by converting it to an amine-reactive NHS ester. This active ester then readily reacts with the primary amine groups (-NH_2_) on lysine residues or on the target molecule (e.g., a peptide’s N-terminus) to form a stable amide bond. This method is extensively used to biofunctionalise SF materials. For instance, the conjugation of cell-adhesive peptides like RGD to SF scaffolds significantly enhances the attachment, spreading, and proliferation of various cell types, including fibroblasts and osteoblasts, for tissue engineering applications [47]. Beyond peptides, EDC/NHS chemistry is crucial for immobilising enzymes for biocatalytic platforms and tethering antibodies to SF-coated sensors and optical fibres to create highly specific biorecognition interfaces for diagnostics [45,46].

Genipin Crosslinking: As a biocompatible and naturally derived alternative to toxic synthetic crosslinkers like glutaraldehyde, genipin is increasingly used to crosslink and functionalise SF. Genipin spontaneously reacts with the primary amine groups (-NH_2_) on lysine residues and the N-termini of SF chains through a two-step mechanism. The reaction initially forms an intermediate heterocyclic amine, which further undergoes a nucleophilic substitution to create stable, blue-pigmented heterocyclic crosslinks. This crosslinking not only renders SF scaffolds water-insoluble and mechanically robust but also does so with a significantly reduced cytotoxicity compared to glutaraldehyde [48]. The reaction can be leveraged for functionalization; molecules containing primary amines can be incorporated during the crosslinking process. Studies have shown that genipin-crosslinked SF hydrogels and films exhibit an excellent biocompatibility and support cell viability, making them particularly suitable for applications in neural tissue engineering and as drug-eluting matrices where a minimal inflammatory response is critical [49,50]. The characteristic blue fluorescence of the genipin–silk reaction product can also be utilised for tracking material degradation in vitro. The precision of chemical conjugation is exemplified in biosensing applications. Antibodies can be covalently immobilised onto the surface of silk-coated optical fibres, creating robust and specific interfaces for the label-free detection of antigens [45]. Similarly, this method enables the functionalization of single silk fibres with fluorophores and sensing elements for advanced imaging and diagnostic platforms [43].

#### 2.6.3. Genetic Engineering and Enzymatic Conjugation Strategies

Genetic- and enzyme-mediated strategies represent the most integrated forms of SF functionalization because they build bioactivity into the protein sequence or modify it under mild, enzyme-catalysed conditions. Unlike post-synthetic chemical coupling, these approaches can provide a highly controlled presentation of functional motifs while preserving overall protein conformation and biocompatibility.

Genetic Engineering: In genetically engineered systems, the SF gene is modified to encode specific bioactive domains (e.g., RGD motifs, BMP-2-mimetic sequences, elastin-like domains, or growth factor-binding peptides) before expression in a recombinant host. This yields silk variants in which functional motifs are distributed with molecular-level precision and at high, uniform density throughout the polymer backbone, eliminating the need for heterogeneous post-production conjugation [51]. As summarised in the review by Aigner et al., recombinant spider- and silkworm-inspired silk proteins fused with RGD, Ile-Lys-Val-Ala-Val (IKVAV), or laminin-derived sequences have been reported to enhance the adhesion, spreading, and differentiation of fibroblasts, osteoblasts, and neurons compared with unmodified silk, while retaining the mechanical robustness and self-assembly behaviour of the parent protein [51]. Genetically encoded mineralisation-directing sequences have also been introduced to promote hydroxyapatite nucleation for bone tissue engineering, and growth factor-binding domains have been fused to silk blocks to localise and stabilise morphogens (e.g., BMP-2, VEGF) within scaffolds. These recombinant designs allow the independent tuning of mechanical properties (via the silk block composition and repeat number) and biological signalling (via the choice and density of fused motifs), offering a level of control that is difficult to achieve with purely chemical modification strategies [52].

Enzymatic Conjugation: Enzymatic conjugation strategies exploit the intrinsic amino acid composition of SF to introduce functional groups through catalytic reactions rather than genetic modification. A well-studied example is the tyrosinase-mediated modification of tyrosine residues in SF. In this approach, tyrosinase oxidises the phenolic side chains of tyrosine to o-quinones, which are highly reactive towards nucleophiles such as primary amines and thiols on target molecules [53]. Subsequent non-enzymatic Michael-type additions or Schiff-base reactions graft chitosan, peptides, or other amine-containing biomolecules onto the silk backbone, without the need for organic solvents or carbodiimide chemistry. Freddi et al. demonstrated that the tyrosinase-catalysed coupling of chitosan to *Bombyx mori* SF films yielded composite materials with enhanced hydrophilicity, swelling, and cell-interaction properties, while preserving the β-sheet structure and mechanical integrity of the silk matrix [53]. Related enzymatic systems using transglutaminase, peroxidases, or laccases have been explored to introduce crosslinks or to immobilise bioactive proteins via glutamine–lysine isopeptide bonds or phenolic coupling, enabling a tighter control over network architecture and biofunctional layer formation on SF surfaces [54].

Overall, genetic engineering and enzymatic conjugation complement physical blending and covalent chemistry by providing sequence-level precision or catalytic site-selective modification routes. Genetic approaches embed bioactivity directly into the silk backbone at the molecular level, whereas enzymatic methods modify already-fabricated structures under controlled aqueous conditions. The choice between these strategies is dictated by the required stability and density of functional groups, the sensitivity of the cargo to chemical conditions, and the intended application—whether for long-term regenerative scaffolds, dynamically responsive hydrogels, or finely tuned biointerfaces for sensing and diagnostics.

**Table 1:** Representative SF morphologies, corresponding processing methods (as described in Section 2), and typical biomedical applications reported in the literature.

Morphology	Processing Method	Application
Fibres and fibrous mats (micro- and nanofibres, non-woven meshes)	Electrospinning/electrospraying	Wound dressings and skin substitutes; tissue engineering scaffolds for skin, nerve, vascular and tendon repair; fibrous carriers for surface or implantable biosensors [52,55,56,57,58,59].
3D porous scaffolds and sponges (including cryogels)	Cryogelation and freeze-drying	Bone and cartilage regeneration; soft-tissue and dermal scaffolds; volumetric defect filling; 3D in vitro cell culture models and guided tissue regeneration [58,60,61,62].
Bulk hydrogels (transparent or opaque gels)	Triggered sol–gel transition	Injectable and in situ-forming scaffolds; cartilage and soft-tissue repair; cell encapsulation matrices; local and sustained delivery depots for drugs, proteins, or cells [2,47,49,54].
Particles and capsules (nanoparticles, microspheres, microcapsules)	Emulsification or desolvation/nanoprecipitation; microfluidics	Controlled and sustained drug delivery; encapsulation and release of bioactive molecules and vaccines; targeted and responsive delivery systems; multifunctional imaging and theranostic carriers [24,41,42].
Thin films and coatings (planar or conformal)	Casting or spin-coating; annealing	Implant and device coatings (orthopaedic, dental, vascular); wound dressings and skin repair matrices; optical and photonic platforms for bioimaging and biosensing; protective biocompatible coatings on nanoparticles and sensors [18,23,63].
Microstructured and lithographically patterned silk devices	Photolithography and soft lithography	Organ-on-a-chip and microfluidic systems; microstructured scaffolds that guide cell alignment and anisotropic tissue growth; all-silk optical elements (waveguides, gratings, resonators) and integrated biosensing devices [23,64].

Although Table 1 summarises the principal silk fibroin morphologies and associated processing strategies, it also highlights important trade-offs that influence application selection. Fibrous systems generated by electrospinning offer a high surface area and ECM-like architecture, making them well suited for wound healing and soft tissue regeneration; however, they may exhibit limited compressive strength and can require post-treatment to stabilise β-sheet content. Porous 3D scaffolds fabricated by salt-leaching or freeze-drying provide a greater structural integrity for load-bearing applications such as bone regeneration, but their pore size distribution and mechanical properties are highly sensitive to porogen size and processing parameters. Hydrogels, while advantageous for cell encapsulation and injectable therapies, often suffer from a lower mechanical robustness and rapid degradation unless chemically or physically crosslinked. Particulate systems allow precise drug loading and controlled release profiles, yet the batch-to-batch variability in particle size and crystallinity can affect release kinetics. Thin films and patterned microstructures offer optical transparency and surface-level functionalisation, but their limited thickness constrains volumetric tissue integration. Hence, the choice of silk morphology is not merely aesthetic or fabrication-driven, but fundamentally dictated by the mechanical demands, degradation timeframe, and mass-transport requirements of the intended biomedical application. A critical understanding of these structure–processing–property relationships is therefore essential for rational scaffold design and successful clinical translation.

## 3. Applications of Silk Fibroin in Biomedicine: From Fundamental Platforms to Integrated Theranostics

### 3.1. Bioimaging

SF functions as an unusually flexible optical biomaterial: it is optically transparent across much of the visible–near-IR window, it is highly biocompatible, and its secondary structure (and therefore mechanical stability and dissolution kinetics) can be programmed through simple post-processing steps such as water-annealing, methanol treatment, or crosslinking [10,18]. These attributes make SF a natural host for fluorophores and luminescent nanoparticles intended for long-term bioimaging and biosensing because silk can both preserve optical performance (by minimising background autofluorescence and scattering) and provide biological functionality (cell support, controlled degradation, and surface chemistry for bioconjugation) that many synthetic matrices cannot. The field that merges SF with bright, photostable emitters spans three complementary directions: (i) nanodiamond (ND) NV-centre hybrids that exploit quantum defects for photostable imaging and temperature sensing [18,31,41,42], (ii) semiconductor quantum dots (QDs) and carbon-based quantum dots (CQDs) integrated with silk for bright, tuneable emission and sensing [65,66,67], and (iii) organic fluorophores (such as fluorescein or Seminaphtharhodafluor (SNARF) dyes) embedded or attached via silk for chemically specific sensing and fibre-optic probe readouts [43,45,68]. Together, these constructs enable long-term cellular tracking, local chemical monitoring, and integrated theranostic applications that are difficult to achieve with other materials.

#### 3.1.1. Silk Fibroin–Nanodiamond Hybrid for Bioimaging

The nitrogen vacancy (NV^−^) centre in fluorescent nanodiamonds (NDs) provides one of the clearest demonstrations of how silk and quantum emitters complement each other. Nitrogen vacancy (NV) centres emit bright, non-bleaching fluorescence and also act as nanoscale sensors of temperature and magnetic fields through optically detected magnetic resonance (ODMR) [31]. However, when NDs are used alone, they suffer from limited photon extraction, surface aggregation, and endosomal trapping [69]. Embedding NDs in silk matrices addresses these challenges. Silk’s refractive index (~1.54) reduces the mismatch between diamond (~2.4) and aqueous environments, improving photon extraction and thus increasing detectable emission [18]. Experimental studies of ND–SF hybrid films and spheres have reported ~1.5–4-fold emission enhancements [18,41]. In vivo murine wound models further confirmed biocompatibility, showing no inflammatory response [18,31]. This establishes silk as both a photonic enhancer and a biologically safe host for quantum emitters.

**Figure 3:**
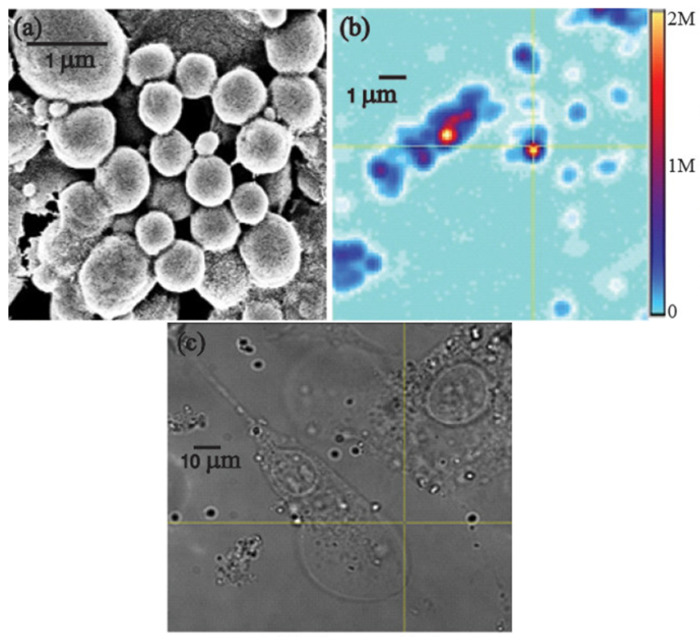
(**a**) Scanning electron microscopy (SEM) image and (**b**) confocal fluorescence map of ND-silk spheres. (**c**) Bright field image of fibroblast cells internalised with the spheres for intracellular imaging. Image reprinted from [41] with permission.

As shown in Figure 3, the cited study demonstrates the fabrication of silk fibroin sub-micron spheres via controlled aqueous processing, resulting in a uniform spherical morphology with a smooth surface topology under SEM imaging. The figure illustrates both the narrow size distribution and structural integrity of the particles following β-sheet induction, confirming successful solidification [41]. Such morphology is critical for predictable drug loading and release kinetics, as the particle diameter directly influences the diffusion length and degradation behaviour. The figure therefore substantiates the discussion in this section regarding how the fabrication route governs the particle size, crystallinity and functional performance in drug delivery systems.

At the sub-micron scale, SF-encapsulated ND spheres produced by aqueous co-flow or nanoprecipitation, shown in Figure 3a,b, offer multifunctional nanocarriers that integrate imaging, therapeutic loading, and controlled biodegradation. These ND–SF spheres, typically within the 300–1000 nm range, exhibit monodispersity and enhanced colloidal stability compared to bare NDs, owing to the amphiphilic nature of SF, which prevents aggregation and minimises nonspecific protein adsorption. Importantly, encapsulation within the silk matrix reduces endosomal sequestration, enabling a higher intracellular mobility and cytoplasmic distribution, as shown in Figure 3c, which in turn facilitates more effective bioimaging and drug delivery [41]. Fluorescence signatures of NV centres—particularly the zero-phonon line at 637 nm and their broad phonon sidebands—remain intact after encapsulation, with emission intensities maintained over extended imaging durations. This structural preservation underscores silk’s role as both a protective shell and an optical stabiliser, ensuring that NV-centre fluorescence remains photostable and non-bleaching, even in biologically complex environments. Furthermore, the intensity variations observed during progressive silk degradation provide a real-time optical readout of carrier stability and degradation kinetics, effectively allowing ND fluorescence to act as a self-reporting probe of the delivery vehicle’s size.

Beyond imaging, therapeutic co-loading demonstrates the dual functionality of ND–SF hybrids. In the case of doxorubicin-loaded ND–SF spheres, silk’s enzymatically degradable β-sheet network serves as a triggerable release mechanism: protease-rich environments accelerate matrix breakdown, thereby enhancing drug release in situ. Simultaneously, changes in NV fluorescence intensity and spectral properties serve as optical reporters of carrier degradation and drug release dynamics [42]. This coupling of long-lived, stable quantum emission with a biodegradable, drug-releasing scaffold represents a unique theranostic paradigm. It enables not only the delivery of therapeutic payloads but also the capacity to optically monitor therapeutic action and carrier degradation in real time, bridging the gap between imaging and therapy. Such ND–SF theranostic spheres exemplify how silk’s biological versatility and ND’s quantum photophysics can be synergistically integrated into platforms for advanced, minimally invasive bioimaging and controlled drug release [42].

#### 3.1.2. Silk–Quantum Dot and Carbon Dot Fluorescent Probes

The optical synergy between silk and embedded emitters is not restricted to NDs. Work that explicitly couples QDs to silk has provided equally compelling outcomes. In a systematic study, SF micro- and nanoparticles were labelled with CdSe/ZnS QDs, and their interactions with cells were evaluated. The results [70] showed a strong particle size dependence: microparticles promoted the adhesion and proliferation of normal endothelial-like cells, while nanoparticles were preferentially internalised by tumour-derived HeLa cells. This size-tuneable uptake, combined with bright emission, suggests that SF–QD hybrids can be tailored either for surface-localised tracking or intracellular delivery. Silk encapsulation has been reported to improve photostability compared with free organic dyes such as fluorescein, and it reduced cytotoxicity relative to uncoated QDs, highlighting silk’s role as both an optical stabiliser and biological shield [70].

Similarly, carbon quantum dots (CQDs) integrated into silk composites further broaden the palette. In one example, CQDs embedded in electroactive silk/PLA nanofibrous scaffolds yielded constructs with enhanced mechanical and swelling properties, bright photoluminescence, and improved cardiomyocyte adhesion and viability [65]. Here, CQDs acted both as fluorescent reporters—enabling the imaging of scaffold colonisation—and as functional additives that promoted tissue regeneration. The biocompatibility of CQDs combined with the versatility of silk scaffolds thus provides a promising route for inherently imageable, multifunctional tissue engineering platforms [65].

The optical interplay between silk and embedded emitters is not merely additive—silk can actively influence performance by reducing aggregation, stabilising surface groups, and preserving quantum yield over extended imaging sessions [18,41]. These stabilising effects apply broadly across NDs, QDs, and CQDs, ensuring brighter and longer imaging windows with less quenching or bleaching than conventional dye–polymer matrices. Despite these optically innovative materials with silk, limitations remain. Nanodiamonds are non-degradable and can accumulate in organs or be internalised by cells after silk sphere degradation [71,72]. QDs carry heavy metal toxicity risks that must be carefully engineered away; and scaling silk processing with consistent β-sheet content and probe distribution is challenging. Nonetheless, silk + fluorescent nanoparticle platforms already show promise for clinically relevant biomaterials for imaging.

### 3.2. Biosensing

SF has emerged as a versatile material platform for biosensing because of its unique combination of optical transparency, mechanical robustness, aqueous processability, and biocompatibility. Its hierarchical β-sheet structure can be tuned to control permeability, rigidity, and degradation, while abundant functional groups provide opportunities for surface modification and probe immobilisation [23,31,43,73]. Unlike many synthetic polymers, silk enables the long-term stability of embedded biomolecules and nanomaterials without the need for harsh processing conditions, which makes it particularly suitable for implantable and wearable sensors in healthcare [17].

#### 3.2.1. Optical Fibre-Based Sensing with Silk Fibroin

The first major advance in this field was reported in [43], which demonstrated the use of SF to stabilise fluorescent probes on exposed core optical fibres for in vivo biochemical monitoring. In this study, the pH-sensitive fluorophore carboxynaphthofluorescein (CNF) was embedded in an SF coating applied to microstructured optical fibres. The silk matrix served as a host that immobilised the dye while preserving its optical response, with the fibre geometry enabling fluorescence coupling into the guided modes for remote detection. A major achievement of this work was the demonstration of continuous in vivo pH monitoring in a mouse model of hypoxia. When the animals were subjected to oxygen deprivation, the silk-coated fibres recorded a progressive decrease in subcutaneous pH, establishing the ability of the platform to capture dynamic physiological changes in real time. The silk coating conferred photostability and reduced dye leaching, while the ratiometric fluorescence readout minimised errors due to variations in probe positioning and excitation conditions. These results confirmed that SF can serve not only as a passive biocompatible coating but also as an active host matrix for sensing elements, enabling durable and accurate optical biosensing in vivo [45,68].

Building on this foundation, the subsequent study [45] extended the capabilities of silk-coated fibres to protein detection. Here, SF was again employed as a biocompatible matrix, but the sensing function was introduced through a silk-binding peptide covalently modified with biotin. By exploiting the strong biotin–streptavidin interaction, the probe was designed to selectively capture fluorescently labelled streptavidin on the fibre surface. The study compared two strategies for incorporating the biotinylated peptide: embedding it throughout the silk coating versus localising it on the surface. The results [45] demonstrated that only surface functionalization enabled efficient streptavidin detection, with a detection limit of approximately 15 µg/mL. Embedding the functionalized peptide throughout the silk matrix significantly reduced sensitivity, likely due to the restricted diffusion of the large protein analyte. This finding underscored the importance of molecular accessibility within the silk layer and provided valuable design principles for adapting silk-based coatings to different classes of analytes. The coatings were further shown to withstand multiple wash cycles and prolonged aqueous exposure, confirming their robustness. While the detection limit remains relatively high compared to advanced protein sensors, this work represents a proof of concept that silk-functionalized optical fibres can be engineered for selective biomolecular recognition, paving the way for applications in diagnostics and biosensing where surface specificity and reusability are required [45].

This line of work culminated in multifunctional sensing and imaging capabilities, with [68] representing a significant advance in this trajectory. In this research, SF was used as a functional coating on an optical fibre tip, enabling both pH sensing and structural imaging. The design employed a silk-binding peptide conjugated to a pH-sensitive dye, which was incorporated into the silk matrix during fibre coating. The resulting construct allowed the precise immobilisation of the sensing molecules, preserving fluorescence stability while reducing dye leaching. Importantly, this fibre not only detected local pH variations but also maintained its capability for optical coherence tomography (OCT) imaging, providing spatial guidance during sensing. In biological demonstrations, the sensor was able to monitor subtle metabolic changes, such as a ~0.04 unit pH drop in the microenvironment of oocytes exposed to cobalt chloride. OCT imaging further enabled the visualisation of ovarian tissue and identification of oocytes within follicles, highlighting the potential of this platform in reproductive medicine. This dual imaging–sensing functionality represents a significant advance for minimally invasive, real-time biochemical monitoring, particularly for applications such as in vitro fertilisation (IVF), where monitoring the microenvironment of developing oocytes is of critical importance. The silk coating was shown to be stable under repeated washing, demonstrating its robustness for biological applications.

#### 3.2.2. Silk-Based Conformal, Adhesive, Edible Food Sensors

**Figure 4:**
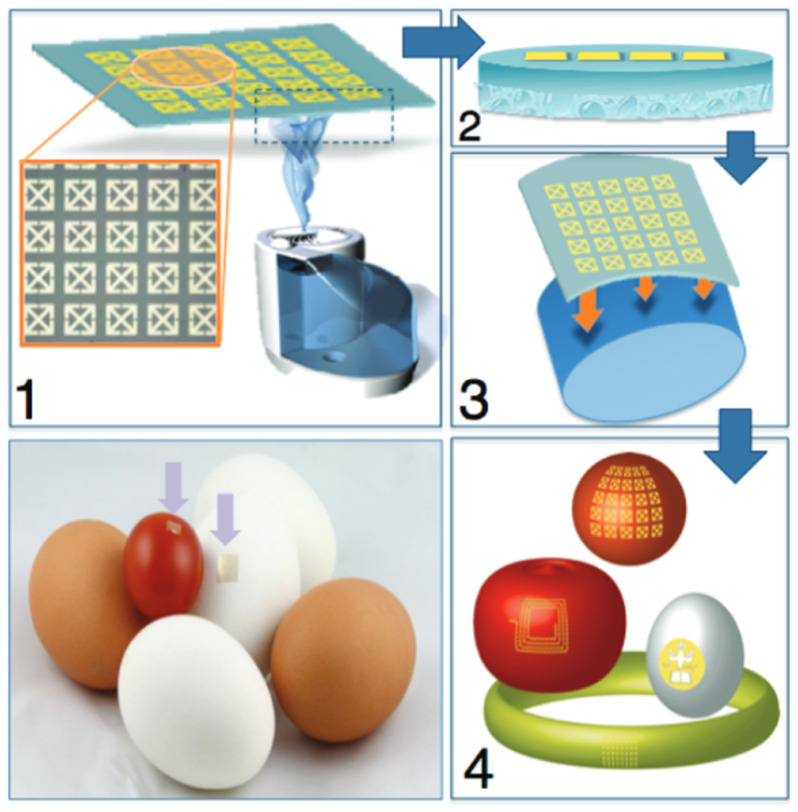
Conformal, adhesive, and edible silk fibroin-based wireless sensing platforms for food monitoring. Regenerated silk fibroin films serve as biodegradable substrates for ultrathin metallic antenna and resonator patterns. Controlled water vapour exposure softens the silk matrix, enabling intimate conformal adhesion to curved food surfaces. In the schematic, the numbered elements indicate: (1) silk fibroin film substrate, (2) resonator/antenna pattern forming a passive LC (inductor–capacitor) circuit, (3) curved food surface, and (4) external reader antenna/interrogation setup. The resulting chip-less, battery-free devices function as passive resonant sensors, with shifts in electromagnetic response arising from changes in the dielectric properties of the underlying food during ripening or spoilage. Reprinted with permission from [74].

The pioneering work reported in [74] marked one of the first demonstrations of SF being utilised beyond traditional biomedical settings to address challenges in food quality and safety monitoring. A representative example of silk-based conformal, adhesive, and edible sensors was reported by Tao et al., who demonstrated wireless, passive sensing platforms fabricated entirely on regenerated silk fibroin films. In this approach, ultrathin gold antenna and resonator structures were patterned onto aqueous-processed silk films using chemical-free transfer and printing methods. Upon exposure to water vapour, the silk substrate softened while retaining structural integrity, enabling an intimate conformal adhesion to highly curved food surfaces such as fruit skins, eggs, and packaged dairy products. The resulting devices function as chip-less, battery-free resonant sensors whose electromagnetic response shifts in response to changes in the dielectric properties of the underlying food during ripening or spoilage. Importantly, the use of pure protein silk substrates and sub-micron gold layers—comparable to edible gold leaf—renders these sensors biodegradable and potentially ingestible, positioning silk fibroin as a unique platform for safe, disposable, and non-destructive food-quality monitoring. A representative example of this concept, illustrating conformal adhesion and wireless passive sensing using silk fibroin films on food surfaces, is shown in Figure 4 [74]. In this study, Tao et al. demonstrate how regenerated silk fibroin can serve as a mechanically compliant and biodegradable substrate for passive wireless sensing. In this system, an ultrathin metallic LC resonator is patterned onto a silk film, which softens upon controlled hydration to conform intimately to curved food surfaces. This conformal adhesion ensures stable electromagnetic coupling, enabling chip-less, battery-free monitoring through resonance frequency shifts that reflect changes in the dielectric properties of the underlying substrate. Unlike enzymatic or colorimetric sensors, the detection mechanism here relies purely on passive electromagnetic response, highlighting silk’s compatibility with flexible electronics and edible sensing platforms.

Subsequent work has extended these concepts by exploiting SF’s ability to form nanoporous, capillary-active architectures for quantitative biochemical sensing. In [75], Marquez and co-workers produced nanoporous silk films doped with glucose oxidase (Gox), HRP, and the chromogenic mediator 2,2′-azino-bis(3-ethylbenzothiazoline-6-sulfonic acid) (ABTS). The films were engineered to exhibit controlled pore sizes and capillary channels that could spontaneously wick microlitre volumes of whole blood while excluding larger components such as red blood cells. This size-exclusion effect minimised fouling and optical interference, enabling the direct glucose determination from whole blood without pre-processing. Compared to solution-phase assays, the nanoporous silk platforms improved sensitivity (reported enhancements of ~2–3×) and demonstrated extended operational lifetimes due to the stabilisation of both enzymes and mediators within the silk network. These findings reinforced SF’s role as a powerful stabilising and mass-transport-modulating matrix for enzymatic colorimetric sensing, relevant not only to medical diagnostics but also to food and beverage quality control where small-volume, complex samples must be analysed rapidly.

Silk has also been engineered into three-dimensional microstructures for food safety applications that go beyond surface monitoring. In [76], Kim et al. developed a porous silk microneedle array capable of sampling fluid from within solid foods and transducing spoilage and contamination signals via colorimetric “bioinks.” The microneedles, moulded from regenerated SF, were mechanically robust and food-safe, piercing packaging and food matrices (e.g., fish fillets, meat, fresh produce) without fragmentation. Capillary action within the porous needle shafts transported interior food fluid to the backside of the patch, where polydiacetylene-based bioinks were printed. These bioinks were designed to change colour in response to pH shifts associated with spoilage and to the presence of pathogenic bacteria such as *Escherichia coli*. In fish fillet models, the sensors could distinguish *E. coli* contamination within ~16 h, with spatially localised colour changes indicating contaminated sites. Importantly, the silk microneedles could pierce commercial plastic packaging, enabling in-pack testing downstream in the supply chain without opening or destroying the product.

Related approaches have applied silk-based sensing directly to seafood products. For example, ref. [77] reports microneedle patches in which SF provides the structural framework and biocompatible interface, while colorimetric chemistries respond to volatile amines and pH changes generated during fish spoilage. These patches showed rapid response times and clear colour transitions that correlated with microbiological and chemical indicators of freshness, allowing real-time spoilage assessment in packaged salmon. The combination of silk’s mechanical robustness, tuneable porosity, and compatibility with food-contact regulations makes such microneedle-based sensors attractive for point-of-use quality testing in retail and domestic settings.

Taken together, these studies [63,74,76,77] collectively establish a set of design principles for silk-based food sensors. SF can be cast as ultrathin edible films, nanoporous capillary matrices, or three-dimensional microneedle arrays; in each case, its β-sheet-stabilised structure preserves the activity of enzymes and chromogenic reporters, while its mechanical and interfacial properties allow intimate contact with food or packaging. These features support the non-invasive, visually readable, and in some cases reusable sensing of key spoilage markers (glucose, pH, volatile amines, bacteria) across the food supply chain, highlighting silk’s unique position as both a functional and sustainable platform for intelligent food monitoring [75].

#### 3.2.3. Silk Fibroin-Based Wearable and Smart Clothing Sensors

SF has also been exploited as a functional material in wearable and textile-integrated biosensors, leveraging its biocompatibility, mechanical flexibility, and compatibility with fibre and fabric processing. In [78], Wen et al. designed an all-fibre sensor architecture in which SF-based yarns formed the dielectric and structural components of pressure, temperature, and humidity sensors integrated directly into textiles. Electrospun SF mats and silk yarns were combined with conductive electrodes (including silver nanowires and conductive threads) to form flexible capacitive and resistive sensing elements that could be woven or stitched into garments. The resulting fabrics demonstrated a high sensitivity to external pressure (e.g., detection of subtle finger tapping and joint motion), reliable temperature sensing in the physiological range, and humidity response relevant to perspiration monitoring. Mechanical testing showed that the silk-based fibre sensors withstood repeated bending, stretching, and laundering cycles with minimal drift, reflecting silk’s intrinsic tensile strength and fatigue resistance. When incorporated into smart gloves and garments, these all-fibre devices enabled the real-time monitoring of joint angles, gait, and respiratory patterns, illustrating how SF can serve both as a comfortable textile substrate and as a key functional component in wearable electronics [78].

Further developments in wearable electrochemical sensing have extended silk’s role from a passive structural material to an active electrochemical substrate. Meng et al. [79] constructed a flexible electrochemical patch by carbonising SF to form N-doped porous carbonised silk fibroin (CSF), which was then loaded with gold nanoparticles (AuNPs) to yield AuNPs@CSF. This hybrid material exhibited intrinsic peroxidase-like “nanozyme” activity, enabling catalytic reduction/oxidation reactions without the need for fragile natural enzymes. By integrating AuNPs@CSF onto flexible electrodes physically crosslinked with polyurethane and SF, the work reports wearable sensors capable of monitoring hydrogen peroxide released from cancer cells and glucose levels in sweat. The devices showed low detection limits within physiologically relevant ranges (on the order of tens of micromolar for H_2_O_2_ and glucose), good linearity over physiologically relevant ranges, and stable performance over at least 30 days of ambient storage, highlighting silk’s capacity to host catalytically active nanostructures while maintaining mechanical compliance with skin.

Complementary strategies have exploited SF nanofibrils and composite films for long-term sweat metabolite monitoring. Liu et al. [80] fabricated a multilayer device in which an SF nanofibril membrane embedded with glucose oxidase and lactate oxidase was laminated onto an ultra-thin platinum nanoparticle/graphene electrode film. Silk’s nanofibrillar network ensured a high enzyme loading, efficient diffusion of sweat analytes, and mechanical conformability to skin, while the PtNP/graphene layer provided a high electrochemical activity. The resulting patch achieved the stable, simultaneous detection of glucose and lactate in sweat with minimal signal drift over extended wear, demonstrating how silk-based enzymatic membranes can be integrated with advanced electrode materials for robust, on-body metabolite monitoring.

**Figure 5:**
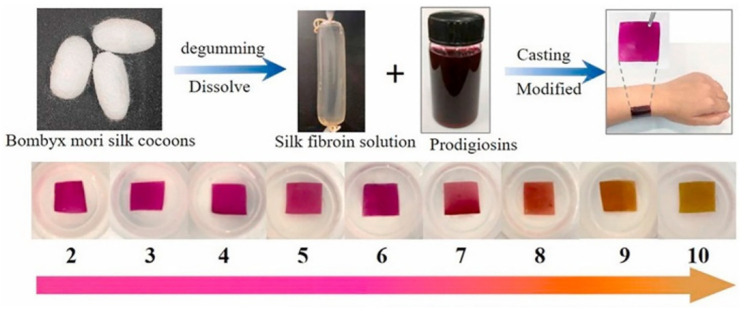
Schematic representation of pH-responsive silk fibroin (SF) composite films incorporating the microbial pigment prodigiosin. Prodigiosin obtained via microbial fermentation is dispersed within regenerated SF solution, followed by casting into flexible films plasticised with glycerol and chemically crosslinked using EDC to enhance mechanical integrity and water stability. The composite films exhibit reversible colour transitions across physiologically relevant pH ranges, shifting from reddish-purple at mildly acidic conditions to orange and yellow under alkaline environments. This reversible response enables conformal, biodegradable colourimetric sensing on skin or food-contact surfaces. Adapted from [81].

In addition to enzymatic and electrochemical formats, SF has also been used as a matrix for pH-responsive colour-changing films based on microbial pigments, enabling colourimetric pH sensing on skin or food surfaces, as demonstrated in Figure 5. In [81], Liu et al. incorporated prodigiosin—a natural red pigment obtained through microbial fermentation—into a regenerated SF solution and cast composite films that display reversible halochromic behaviour over alkaline pH ranges. Figure 5 summarises the material design strategy and sensing mechanism. The schematic illustrates the integration of the bio-derived pigment within the regenerated SF matrix, followed by plasticisation and carbodiimide-mediated crosslinking to stabilise the composite network. The figure highlights the reversible chromatic transition across different pH values and emphasises the conformal film geometry suitable for direct contact with biological or food surfaces [81]. This visual representation supports the discussion that silk fibroin can host both natural pigments and synthetic dyes while maintaining mechanical flexibility, environmental responsiveness and biodegradability. As shown in Figure 5, the resulting SF–prodigiosin films showed a distinct colour transition from reddish-purple (λ_max_ ≈ 530–535 nm) under mildly acidic to neutral conditions (pH < 8) to orange and finally yellow (λ_max_ ≈ 480 nm) as the pH increased toward 10. To tailor mechanical robustness and water interaction, glycerol was introduced as a plasticiser and EDC as a crosslinker; this combination markedly decreased the water contact angle (from ~83° to ~5°) and improved the flexibility and toughness of the films while preserving their optical response. The authors demonstrated that these SF–prodigiosin films are biodegradable, biocompatible and can adhere to skin for prolonged periods, enabling long-term, visual pH indication on soft biological or food-contact surfaces. As illustrated in Figure 5, this design exemplifies how silk’s film-forming ability, tuneable secondary structure and compatibility with natural pigments can be harnessed to create conformal, colourimetric platforms suitable for wearable pH sensing, intelligent packaging and other stimulus-responsive biosensing applications [81].

Broader perspectives on these developments are provided by the review [82], which surveys SF and sericin as substrates and active components in wearable sensors for electrocardiogram (ECG), electromyography (EMG), pulse, respiration, temperature and biochemical monitoring. Across these systems, silk-based fibres, films and coatings are repeatedly exploited for their breathability, conformal skin contact, and ability to stabilise functional nanomaterials and biomolecules under mechanical deformation. Taken together, the studies discussed in this subsection position SF as a core material for next-generation wearable electronics and smart textiles, in which the same protein network provides mechanical comfort, long-term skin compatibility and a versatile host for electrochemical, optical and colorimetric transduction. In the following subsection, we focus more specifically on silk-based colorimetric platforms, where reusability and sustainability are key design drivers.

#### 3.2.4. Reusable Colorimetric Biosensors on Sustainable Silk-Based Platforms

In another important study [63], the authors demonstrated how SF can underpin sustainable, reusable colorimetric biosensors designed to replace conventional single-use plastic-based test strips. In this work, SF was processed into thin, optically transparent films that were photopatterned with an enzymatic and photochromic sensing system. The films incorporated GOx and horseradish peroxidase (HRP) as the enzymatic core, together with dithienylethene (DTE) photochromic molecules acting both as colour mediators and photopatternable elements. By exploiting DTE’s reversible open–closed isomerization, the authors were able to generate spatially defined coloured regions on the silk films using light, effectively “printing” biosensor patterns without additional masks or inks. Upon exposure to glucose, the GOx/HRP cascade produced hydrogen peroxide, which triggered DTE-mediated colour changes visible to the naked eye. Silk’s β-sheet-stabilised network immobilised both enzymes and photochromic molecules, preventing leaching and preserving activity under room-temperature storage for up to several months. The films could be washed repeatedly and reused with limited loss of signal, illustrating how SF enables both functional stability and reusability in colorimetric biosensing.

Silk’s compatibility with in situ metal nanoparticle formation has also been leveraged to create highly sensitive, broad-range colorimetric sensors for toxic metal ions. Mane et al. [73] synthesised silver nanoparticles directly within a *Bombyx mori* SF matrix, using silk both as a reducing agent (via tyrosine residues) and as a stabilising scaffold. The resulting AgNP–silk nanocomposite dispersion exhibited a strong surface plasmon resonance (SPR) band, the position and intensity of which shifted upon interaction with Hg^2+^ ions. By casting the dispersion onto solid substrates to form interfacial films, the authors created solid-phase colorimetric sensors capable of detecting mercury at parts-per-billion (ppb) levels through visible colour changes. The silk matrix stabilised the AgNPs against aggregation, extended shelf life, and provided a biocompatible interface suitable for environmental and water-quality testing. This work highlighted SF’s potential as a green, bio-based template for noble metal nanoparticle synthesis and as a functional platform for heavy metal detection.

Related approaches further diversify the analyte scope and optical mechanisms within silk-based sensing systems. In [83], Sanjeevappa et al. reported the in situ synthesis of silver nanoparticles (AgNPs) within silk sericin under microwave irradiation, where tyrosine residues in sericin reduced Ag^+^ ions to metallic Ag^0^. The resulting sericin–AgNP nano-biocomposite exhibited a characteristic surface plasmon resonance (SPR) band at 412 nm and was used as a colourimetric optical sensor for Hg^2+^ detection in aqueous solution. The addition of mercury ions led to a marked decrease in SPR intensity and a visible color change from yellow to colourless, enabling sensitive detection down to 15 ppb. Although based on silk sericin rather than fibroin, this study demonstrates how silk protein chemistry can facilitate green nanoparticle synthesis and provide a biocompatible matrix for plasmonic sensing applications.

Beyond metal-ion and glucose detection, SF films functionalised with natural pigments and redox dyes also fit within this class of reusable colorimetric platforms. The SF–prodigiosin pH-responsive films (discussed in Section 3.2.4 [81]) illustrate how a microbial halochromic pigment can be stabilised within a silk matrix and exhibit reversible colour transitions across physiologically and environmentally relevant pH ranges. Together with [63] and AgNP-based heavy metal sensing systems, these examples demonstrate that both synthetic and bio-derived chromophores can be immobilised in SF to yield biodegradable, visually interpretable colorimetric platforms for monitoring pH, metabolites and toxic metals.

Overall, these colorimetric systems—spanning enzymatic DTE-based glucose sensors, AgNP–silk films for Hg^2+^ detection, fluorescent AgNP–silk probes for Cu^2+^/pyrophosphate, and pigment-doped pH-responsive films—illustrate the breadth of analytes and optical transduction mechanisms that can be implemented on SF platforms. Silk’s ability to immobilise dyes, enzymes, and nanoparticles within a stable β-sheet network underpins its reusability and long shelf life, while its biodegradability and edibility address the environmental burden of disposable plastic-based sensors. Consequently, SF-based colorimetric biosensors represent a convergence of high analytical performance, visual readability, and sustainability, positioning silk as a key material in the transition toward greener diagnostic and monitoring technologies [63].

### 3.3. Silk Fibroin in Wound Healing, Wound-Sensing and Local Drug Delivery

**Figure 6:**
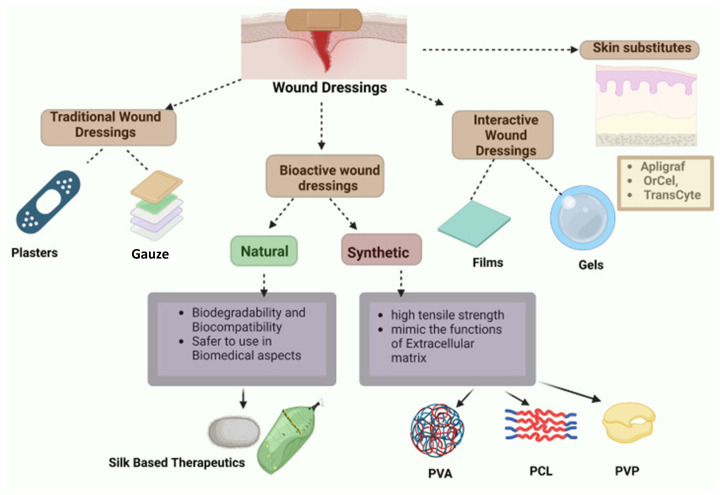
Schematic classification of wound dressings and the position of SF-based bioactive dressings within this landscape. Traditional dressings (e.g., gauze, plasters) mainly provide physical protection, whereas interactive dressings (films, gels, foams) help maintain a moist environment and support re-epithelialisation. Bioactive dressings include natural and synthetic materials that can deliver therapeutic agents and actively modulate the wound microenvironment; silk-based systems fall within this bioactive class, combining biodegradability and biocompatibility with the ability to incorporate growth factors, antimicrobials and sensors. Adapted from [85].

SF has emerged as one of the most versatile protein-based biomaterials for cutaneous repair, functioning both as a passive scaffold that supports cell migration and as an active component in advanced “smart” dressings that integrate sensing, antimicrobial, and drug delivery functions. Its mechanical robustness, excellent oxygen and water vapour permeability, and the ability to tune degradation via β-sheet content make it suitable for both acute and chronic wounds. Recent systematic and mechanistic reviews highlight that SF-based dressings can be processed into films, foams, nanofibrous meshes, hydrogels, and bioadhesives and can be functionalized with antibiotics, growth factors, natural products, and nanomaterials to endow antibacterial and anti-inflammatory properties [5,84,85]. In particular, the review in [85] emphasises the potential of electrospun SF nanofibres as bioactive dressings that support angiogenesis, re-epithelialisation, and extracellular matrix remodelling in impaired diabetic wounds. Importantly, silk-based dressings have progressed from in vitro testing to animal studies and clinical evaluation trials for partial- and full-thickness skin wounds, demonstrating both safety and clinical efficacy [86]. An overview of how SF-based bioactive dressings fit within the broader classification of wound dressings is shown in Figure 6. The figure presents a hierarchical classification of wound dressings and situates silk fibroin-based systems within the “bioactive dressing” category. The schematic contrasts traditional passive dressings, which primarily provide mechanical protection, with interactive and bioactive platforms that actively modulate the wound environment [85]. In the referenced work, electrospun silk fibroin nanofibres are highlighted as representative bioactive matrices capable of mimicking extracellular matrix architecture, supporting cell adhesion and proliferation, and serving as reservoirs for therapeutic payloads. The figure further illustrates how SF-based dressings can integrate antibacterial agents, growth factors and anti-inflammatory compounds, thereby combining structural support with controlled local drug delivery. By positioning silk within this broader framework of wound care technologies, the figure reinforces the argument that SF dressings function not merely as protective coverings but as multifunctional regenerative platforms that bridge scaffold design and therapeutic intervention.

#### 3.3.1. Nanodiamond–Silk Electrospun Membranes as Theranostic Wound Dressings

**Figure 7:**
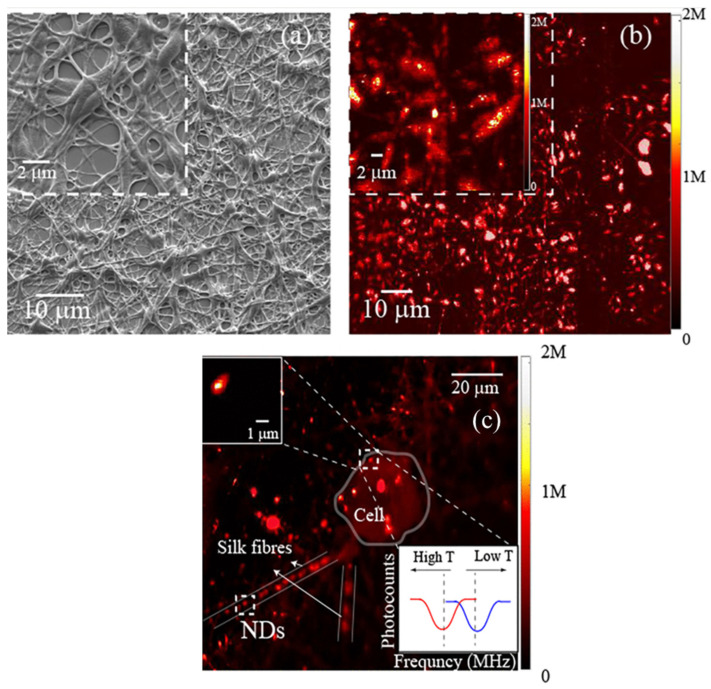
(**a**) SEM and (**b**) 50 × 50 μm^2^ confocal fluorescence map of a post-methanol-treated ND–silk membrane deposited on a glass cover slip. The SEM and confocal fluorescence images show different areas of the same sample. The insets inside the dashed white boxes show higher magnification scans of the samples both for SEM and confocal fluorescence images. (**c**) Human skin keratinocyte (HaCaT) cells grown on electrospun membranes, where the fluorescence of NDs can track local temperature changes via ODMR technique (inset) [31]. Image reprinted with permission.

An innovative work by Khalid et al. reported electrospun nanodiamond–SF membranes [31], which represent a clear example of how silk can bridge wound healing with local, high-resolution sensing. In this study, nanodiamonds (NDs) containing nitrogen vacancy (NV^−^) centres were embedded in electrospun SF nanofibres (as demonstrated in Figure 7a,b) to yield conformable, sub-micron fibrous mats that simultaneously act as wound dressings and optical sensing platforms. The electrospinning process produced a porous, ECM-like architecture that supports cell attachment and nutrient diffusion, while the uniformly distributed NDs preserved their characteristic NV^−^ emission and optically detected magnetic resonance (ODMR) signatures across physiologically relevant temperatures (25–50 °C). Compared with bare FNDs in aqueous suspension, the silk matrix improved photon collection efficiency and thermometric sensitivity by leveraging refractive index matching between the diamond core and the biological environment.

From a sensing perspective, the ND–silk membranes enabled non-invasive, light-based temperature mapping via shifts in the ODMR frequency, providing a local readout of wound surface temperature with high sensitivity, as shown in Figure 7c. The figure provides a structural and functional validation of the nanodiamond–silk theranostic platform. Figure 7a,b confirm the successful incorporation and spatial distribution of fluorescent nanodiamonds within the electrospun silk matrix, demonstrating uniform fibre morphology under SEM and preserved NV^−^ fluorescence under confocal mapping. The higher-magnification insets reveal the homogeneous embedding of the nanodiamond particles within individual nanofibres, indicating that the electrospinning and post-methanol treatment steps do not compromise the fibre integrity or quantum emission properties. Figure 7c further illustrates the biological interface, showing keratinocyte adhesion and proliferation on the fibrous membrane while simultaneously enabling optically detected magnetic resonance (ODMR)-based temperature readout. The inset demonstrates the spectral shift used for thermometry, directly linking material architecture to functional sensing capability. Together, the figure substantiates the dual role of the composite membrane as both a regenerative scaffold and a quantum-enabled wound monitoring device.

In this work [31], since temperature is a key indicator of inflammation and infection, this capability directly links the material’s quantum photonic properties to clinically relevant parameters. At the same time, in vitro assays showed that the membranes supported fibroblast adhesion and proliferation, and in vivo experiments in murine full-thickness skin wounds demonstrated wound closure and tissue regeneration comparable to or better than control dressings, with no evidence of adverse inflammatory responses. The incorporation of NDs also endowed the dressings with selective antibacterial effects against Gram-negative species while maintaining a good cytocompatibility with mammalian cells. Together, these findings show that silk can host non-degradable quantum emitters in a way that preserves their sensing function and simultaneously provides a mechanically and biologically suitable wound interface.

#### 3.3.2. Silk Fibroin Dressings for Accelerated Wound Healing

Beyond nanodiamond-based systems, a large body of work has established SF as a stand-alone wound dressing material in both preclinical and clinical contexts. SF films, sponges, and nanofibres have been shown to support the adhesion and proliferation of keratinocytes, fibroblasts, and endothelial cells, while modulating inflammatory responses and extracellular matrix (ECM) deposition [87]. For example, Naik et al. reported the clinical evaluation of collagen–silk fibroin membranes applied in wound management, demonstrating favourable epithelial coverage, moisture balance, and patient comfort in a case series setting [86]. Although not a randomised controlled trial, the study provided preliminary clinical evidence supporting the biocompatibility and regenerative potential of silk-based composite membranes in cutaneous repair. Such findings highlight the translational relevance of silk fibroin systems while underscoring the need for larger, controlled clinical studies to establish comparative efficacy [86].

At the architectural level, nanofibrous silk matrices generated by electrospinning exhibit a high porosity and specific surface area, mimicking the native dermal ECM and promoting haemostasis and cell infiltration [88]. The incorporation of bioactive agents into these fibrous scaffolds further accelerates healing. For instance, a propolis-enriched SF–gelatin composite nanofibre dressing improved re-epithelialization, granulation tissue formation, and collagen organisation in full-thickness wounds, supported by both in vitro and in vivo analyses [89]. Growth factor delivery has also been effectively implemented: heparinized SF hydrogels loaded with fibroblast growth factor-1 (FGF1) provided a sustained growth factor release, enhanced angiogenesis, and significantly improved wound closure in rat models relative to controls [87]. In diabetic wound models, insulin-loaded SF microparticles or microneedle systems have been shown to stabilise hypoxia-inducible factor-1α (HIF-1α), thereby promoting angiogenesis, ECM deposition, and re-epithelialization [90]. Collectively, these studies indicate that SF not only provides a structural scaffold but also serves as an effective depot for growth factors and therapeutics that correct impaired healing pathways in chronic wounds.

#### 3.3.3. Antibacterial and Immunomodulatory Silk Fibroin Composites

A major challenge in wound management is controlling infection without inducing cytotoxicity or delaying healing. SF-based dressings have been extensively functionalized with antibacterial agents—both inorganic (e.g., silver, zinc oxide) and organic (antibiotics, natural extracts)—to achieve this balance [84]. A representative example is the unilateral silver-loaded SF difunctional membrane developed by Shao et al. [91]. In this design, silver nanoparticles were selectively loaded onto one side of an SF membrane through a simplified layer-by-layer technique, creating an “Ag-rich” face with strong antibacterial activity and an “Ag-free” silk surface optimised for cell contact. In vitro assays showed that both sides exhibited antibacterial effects, but the silk-facing side supported a higher cell viability and up-regulated the expression of collagen I and transforming growth factor (TGF)-β, indicating favourable conditions for tissue regeneration. By confining most of the silver to one surface, this unilateral architecture broadened the therapeutic window of Ag-based treatments, reducing cytotoxicity while maintaining bactericidal performance [91].

More complex nanocomposite hydrogels have also been engineered. Yan et al. reported an SF hydrogel incorporating silver-decorated reduced graphene oxide (Ag@rGO) and poly(γ-glutamic acid), yielding a dressing with combined antioxidant and photothermal antibacterial capabilities [92]. Under near-infrared irradiation, the hydrogel generated localised hyperthermia sufficient to kill bacteria, while its antioxidant properties mitigated oxidative stress in the wound bed. In vivo, this composite promoted the rapid closure of full-thickness, bacteria-infected wounds, enhanced angiogenesis, and stimulated collagen deposition, demonstrating that silk can act as a mechanically stable and biocompatible host for multimodal antimicrobial strategies. Other studies have used antibiotic-loaded silk films for burn wound healing or combined silk with collagen [86], chitosan, and other biopolymers to tailor mechanical properties and biological responses [91,93,94,95].

#### 3.3.4. Towards Smart and Responsive Silk Fibroin Wound Dressings

The integration of sensing capabilities directly into wound dressings is becoming increasingly important for managing complex wounds, where the early detection of infection or imbalance (e.g., excessive protease activity, hypoxia) can significantly improve outcomes. The ND–silk electrospun membranes already exemplify this concept by providing local temperature sensing on a flexible, bioactive scaffold [96]. Complementary approaches, summarised in recent reviews on smart dressings, have used SF in combination with enzyme-cleavable peptides, pH-responsive dyes, or conductive components to create dressings that respond dynamically to the wound microenvironment [97]. For example, gelatinase-cleavable antimicrobial peptides embedded in silk-based networks have been used to produce “on-demand” antibacterial action, where peptide release and structural changes are triggered by elevated matrix metalloproteinase levels in chronic wounds [5,97]. In parallel, conductive silk-based composites are being explored as platforms for electronic wound monitoring, taking advantage of silk’s compatibility with flexible electrodes and microfabrication techniques [5].

Mechanistic studies on the cutaneous regeneration mechanism of β-sheet-rich SF further support the design of such smart systems by clarifying how silk structure influences cell behaviour and immune responses [98]. β-sheet-rich silk has been shown to promote re-epithelialization, modulate macrophage polarisation, and support organised collagen deposition, all of which are critical for functional skin regeneration. When combined with local environment-responsive elements [31], enzyme-cleavable linkers, or pH-sensitive fluorophores, these intrinsic biological effects can be coupled to informative readouts, yielding true theranostic dressings that both guide and report on the healing process. Recent work also suggests that silk-based dressings can be tailored to specific wound types, including venous and arterial ulcers, diabetic foot lesions, third-degree burns, and neoplastic ulcers, by adjusting the formulation, incorporated actives, and degradation kinetics [84].

Taken together, these studies position SF as a central material in the evolution from passive wound covers to active, information-rich wound care systems. From electrospun ND–silk membranes capable of quantum-level thermometry, through growth factor- and insulin-loaded hydrogels and microparticles for chronic wound repair, to silver- and graphene-based nanocomposites with photothermal antibacterial effects, SF consistently provides the structural, chemical, and biological framework needed for safe and effective wound management. As fabrication and functionalization strategies continue to mature, silk-based dressings are likely to underpin the next generation of smart wound interfaces that simultaneously support regeneration, control infection, and deliver clinically actionable information about the wound microenvironment.

### 3.4. Silk Fibroin in Tissue Engineering, Regenerative Medicine and Drug Delivery

SF has become one of the most extensively investigated natural polymers for tissue engineering, owing to its unusual combination of mechanical robustness, tailorable degradation, processability into diverse 3D architectures and excellent cytocompatibility. Early foundational work by Kaplan, Omenetto and co-authors helped frame SF as a “platform biomaterial” in which optical, mechanical and biological functions can be engineered simultaneously, positioning SF as a structural scaffold and an active microenvironment for tissue regeneration [17]. Other subsequent studies from around the globe have defined silk’s role across musculoskeletal, vascular, neural and ocular applications, and have demonstrated that the scaffold architecture, β-sheet content and composite formulation can be tuned to match the functional demands of specific tissues. Comprehensive reviews now consistently place SF among the most promising scaffold biomaterials for regenerative medicine, emphasising its capacity to support cell adhesion and differentiation while maintaining mechanical integrity during tissue formation [56].

A major area where SF has shown clear advantages is bone tissue engineering. Mandal et al. [61] demonstrated that the fabrication route critically determines the mechanical performance and regenerative potential of SF scaffolds. In high-strength silk protein scaffolds for bone repair, they used hexafluoroisopropanol (HFIP) casting and salt leaching to generate highly porous yet mechanically robust 3D silk scaffolds, achieving compressive properties approaching those of trabecular bone while maintaining a high porosity for cell infiltration. Human mesenchymal stem cells (hMSCs) seeded into these scaffolds underwent osteogenic differentiation in vitro and supported new bone formation in vivo in critical-sized rat femoral defects, confirming that silk alone—without ceramic reinforcement—can provide both structural support and an osteoconductive environment. Complementary work on premineralised silk scaffolds showed that in vitro apatite deposition on SF matrices further enhanced osteogenic gene expression and mineralised matrix deposition compared with non-mineralised silk, highlighting the importance of integrating mineral phases for load-bearing applications [99].

Anisotropy and structural hierarchy are central features of native bone, and silk’s processability has been exploited to reproduce these features. Oliveira et al. used the controlled freeze-casting of SF solutions to generate lamellar, longitudinally aligned pore architectures that mimic osteonal lamellae [58]. By adjusting solute concentration and freezing conditions, they produced scaffolds with tuneable lamellar spacing and mechanical anisotropy. Osteoblast-like cells cultured on these lamellar scaffolds aligned along the pore direction and deposited an oriented extracellular matrix, demonstrating that silk’s architecture can provide “contact guidance” cues that are directly relevant to bone regeneration. Systematic and mechanistic reviews of silk scaffolds for bone and musculoskeletal repair have reinforced these findings, consolidating evidence that SF architectures can be tuned to achieve adequate compressive strength, controlled degradation and support for osteogenesis in a variety of preclinical models [100].

SF has also been widely used in cartilage and osteochondral tissue engineering. Through in vitro cartilage tissue engineering with 3D porous aqueous-derived silk scaffolds and mesenchymal stem cells, Wang et al. processed SF using entirely aqueous methods to produce salt-leached, highly porous scaffolds that avoided organic solvents and HFIP processing [62]. Human MSCs seeded within these scaffolds and cultured in a chondrogenic medium formed hyaline-like cartilage, with abundant glycosaminoglycan (GAG) deposition and type II collagen expression. Mechanical testing indicated that the constructs gained equilibrium modulus over time, reflecting matrix accumulation and functional maturation. Follow-up studies have combined SF with cartilage extracellular matrix (ECM) components or gradient mineralisation to produce biphasic scaffolds supporting both cartilage and subchondral bone regeneration in osteochondral defect models [101]. These works underscore silk’s ability to serve as a base polymer that can be combined with ECM cues and spatial patterning to guide site-specific tissue formation.

**Figure 8:**
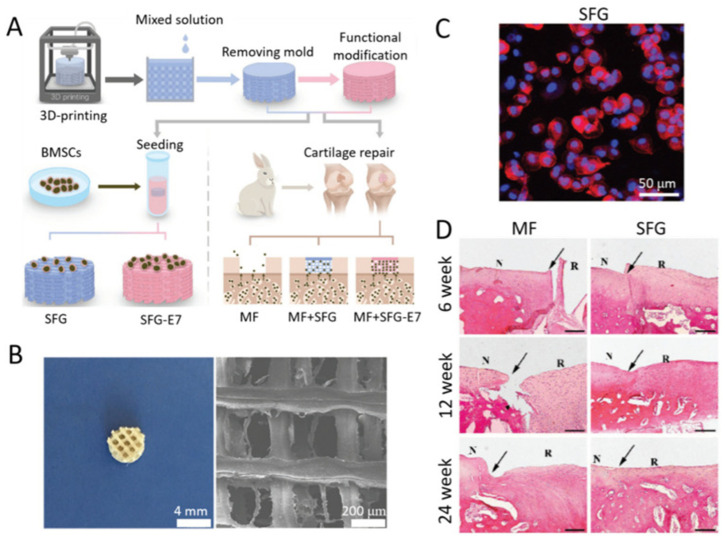
3D-printed silk fibroin–gelatin (SFG) scaffold for cartilage repair. (**A**) Schematic illustration of bioprinting and implantation of the printed scaffold into a cartilage defect. (**B**) Optical microscopy and scanning electron microscopy (SEM) images showing the porous, interconnected architecture of the printed scaffold (SF:gelatin mass ratio 1:2, as reported in the source study). (**C**) Fluorescence staining of bone marrow-derived mesenchymal stem cells (BMSCs) cultured on the silk fibroin–gelatin scaffold after 21 days (phalloidin: F-actin/cytoskeleton; Hoechst: nuclei). (**D**) Hematoxylin and eosin (H&E) staining of repaired cartilage at 6, 12, and 24 weeks post-implantation, comparing microfracture (MF) controls with regenerated cartilage (R) and normal cartilage (N); black arrows indicate the margin between repaired and native cartilage (scale bar: 200 μm). Reproduced from [102].

Beyond bone and cartilage, Mauney et al. have demonstrated that silk’s structural and biochemical context can be tailored to support a wide range of soft tissues. By engineering adipose-like tissue in vitro and in vivo utilising human bone marrow and adipose-derived mesenchymal stem cells with SF 3D scaffolds, 3D porous SF constructs were used to support adipogenic differentiation of both bone marrow- and adipose-derived MSCs [60]. Under adipogenic conditions, cells within silk scaffolds accumulated lipid droplets and expressed adipose markers in vitro, and when implanted subcutaneously, the constructs developed a stable, vascularised adipose-like tissue. These results [60] illustrated that the same basic silk fabrication platform could be re-purposed for different tissue lineages by altering biochemical cues, without changing the underlying protein backbone. Related work has extended SF scaffolds to tendon/ligament and enthesis repair, where braided or woven silk constructs—sometimes combined with silk–gelatin microsponges or mineralised interfaces—provide high tensile strength and gradated mechanical properties, supporting fibroblast infiltration and collagen deposition in preclinical models [55]. A representative example of a 3D-printed SF-based scaffold for cartilage repair is shown in Figure 8.

Figure 8 (reproduced from [102]) provides a representative example of a 3D-printed silk fibroin–gelatin scaffold applied to cartilage repair, linking the fabrication workflow with structural and biological outcomes. Figure 8A summarises the bioprinting and implantation approach, while Figure 8B shows an interconnected porous architecture (optical microscopy and SEM) that supports nutrient transport and cell infiltration. Figure 8C illustrates BMSC attachment and cytoskeletal organisation on the scaffold after 21 days in culture. Figure 8D presents histological evaluation over 6–24 weeks, comparing microfracture controls with regenerated and native cartilage and showing progressive defect filling and improved tissue morphology. Together, the figure highlights how the 3D printing of silk-based composite bioinks enables a controlled scaffold architecture and supports cartilage regeneration in vivo.

Vascular and neural tissue engineering represent further domains where silk’s mechanical and degradation profiles have been leveraged. Lovett et al. fabricated tubular SF scaffolds as small-diameter vascular grafts, demonstrating a sutureable strength, controlled porosity and haemocompatibility in vitro and in vivo [57]. Subsequent preclinical studies have evaluated silk-based grafts for arterial and venous replacement, reporting an encouraging patency, endothelialisation and remodelling, and suggesting that silk’s slow resorption can provide a temporary mechanical template while neovessels form [103]. In peripheral nerve repair, both “silk-in-silk” conduits and composite SF nerve guidance channels have been designed to provide aligned luminal structures and the controlled release of neurotrophic factors. These constructs have achieved regeneration outcomes comparable to autografts in rodent models, with improved axonal alignment and vascularisation across nerve gaps [59]. Collectively, these examples show that silk’s tensile strength, toughness and controlled degradation profiles can be matched to mechanically demanding tissues while preserving a permissive environment for reinnervation or revascularisation.

Ocular tissue engineering has been another important focus area for silk-based biomaterials. In silk film biomaterials for cornea tissue engineering, Lawrence et al. designed ultra-thin (≈2 µm) SF films to mimic the anisotropic lamellar collagen architecture of the corneal stroma while maintaining a high optical transparency [104]. Surface topographies were patterned to direct corneal stromal cell alignment, and the films supported the proliferation and ECM deposition of corneal fibroblasts and epithelial cells, without eliciting inflammatory responses in vitro. By tuning the β-sheet content, degradation rates were adjusted to match the slow remodelling of corneal tissue. These findings, together with subsequent work on optically active silk films and waveguides, established the principle that silk scaffolds can be simultaneously transparent, mechanically stable, and biologically functional, a combination that remains challenging for many synthetic polymers [104].

More recently, advances in additive manufacturing and 3D printing have further expanded the design space for silk-based tissue engineering scaffolds. 3D-printed SF inks, sometimes blended with ceramics or other biopolymers, have been used to fabricate patient-specific bone grafts and complex lattice architectures with gradient porosity and mechanical properties [105]. These approaches allow a more precise control over pore size, interconnectivity and stiffness than traditional salt-leaching or freeze-drying, and can incorporate the spatial patterns of growth factors or ECM components to mimic tissue heterogeneity. Concurrently, emerging data on silk bioinks, hybrid composites and gene-functionalised silk variants underscore a shift from simple porous scaffolds towards highly engineered, multi-functional constructs [52].

Hence, SF is not a single, fixed scaffold material but a family of tuneable matrices that can be adapted to the mechanical, structural and biological requirements of very different tissues. By varying processing routes (aqueous vs. HFIP-based), porogen type, directional freezing, mineralisation and composite formulation, SF scaffolds have been engineered for cartilage, bone, adipose, ligament, nerve, vascular and corneal regeneration, with consistent evidence of good cytocompatibility and constructive remodelling in vivo. The current challenges include standardising silk processing to reduce batch-to-batch variation in β-sheet content and degradation, integrating vascularisation and innervation cues into larger constructs, and translating preclinical successes to controlled clinical trials. Nonetheless, the trajectory of the field—from early porous scaffolds to architected, tissue-specific, and even 3D-printed silk systems—indicates that SF will remain a central biomaterial in tissue engineering and regenerative medicine.

#### 3.4.1. Additional Composite and Tissue-Specific Regenerative Platforms

Beyond the scaffold and depot systems discussed above, recent work has expanded silk fibroin (SF) into more complex composite architectures that integrate bioactive polymers, small molecules, or tissue-derived matrices to enhance regenerative specificity while preserving structural control. Hybrid hydrogel systems incorporating SF microparticles within carboxymethyl cellulose (CMC) matrices have been explored as advanced wound dressings and injectable therapeutic platforms. For example, SF microparticle–CMC composite gels demonstrated an improved mechanical stability and sustained release of loaded antibiotics compared with single-network hydrogels, while maintaining a high moisture retention and oxygen permeability in wound models [106]. In these systems, CMC contributes rapid swelling and exudate management, whereas β-sheet-stabilised SF microparticles provide structural reinforcement and controlled drug release, illustrating how degradation kinetics can be spatially and temporally decoupled within hybrid networks.

Similarly, SF has been combined with hyaluronic acid (HA) and antioxidant agents such as curcumin to modulate inflammatory microenvironments and enhance tissue repair. Curcumin-loaded SF/HA scaffolds have demonstrated reduced oxidative stress and improved re-epithelialization in chronic wound models [107]. The incorporation of curcumin within the silk matrix improves its stability and enables sustained local release, while HA enhances cell adhesion and migration via CD44-mediated pathways. These systems highlight SF’s role as both a stabilising reservoir for labile therapeutics and a mechanically robust extracellular matrix analogue.

In parallel, hybrid electrospun scaffolds combining *Bombyx mori* SF with recombinant spidroins have been developed to enhance tensile strength and elasticity for mechanically demanding tissues such as peripheral nerve and skin wounds [95,108]. These hybrid silk systems exploit complementary β-sheet architectures from distinct silk families, reinforcing mechanical resilience without compromising biodegradability.

Collectively, these composite strategies reflect a broader trend toward hierarchical, multifunctional protein biomaterials in which SF functions as a structural and biochemical integrator rather than a passive scaffold.

#### 3.4.2. Growth Factor Delivery, Neuroregeneration and Advanced Porous Architectures

The controlled delivery of growth factors and neurotrophic molecules from SF matrices represents an important extension of silk’s regenerative role. Owing to its mild aqueous processing and tuneable β-sheet stabilisation, SF preserves protein bioactivity while enabling a sustained release governed by the crystallinity and degradation rate [35,56]. Biodegradable SF scaffolds incorporating glial cell line-derived neurotrophic factor (GDNF) and other neurotrophic cues have been investigated for nerve regeneration, demonstrating an enhanced axonal growth and functional recovery in preclinical models [109]. More broadly, SF microspheres and hydrogels have been employed for the controlled delivery of insulin, fibroblast growth factor (FGF), vascular endothelial growth factor (VEGF), and chemotherapeutic agents, with release profiles directly modulated by the β-sheet content and crosslinking density [36]. These studies reinforce the central structure–property relationship underlying silk-based regenerative systems: mechanical stability and biodegradation must be co-optimised to achieve predictable therapeutic output [6,10].

Advances in processing strategies have expanded SF into ultralight aerogel formats fabricated via freeze-drying or supercritical drying. Silk fibroin aerogels exhibit a high porosity, large internal surface area, and low density, making them attractive for bone regeneration and volumetric tissue reconstruction [110]. When combined with hydroxyapatite or bioactive glass, these aerogels demonstrate improved compressive properties and osteogenic potential. Although largely at the preclinical stage, these architectures illustrate how the extreme structural tuning of SF can extend its applicability into load-bearing regenerative contexts [111].

Together with the scaffold systems discussed earlier in this section, these emerging composite and growth factor-integrated platforms underscore silk fibroin’s versatility across regenerative medicine. While Section 3.1, Section 3.2 and Section 3.3 highlight optical and sensing-enabled constructs, the systems described here reinforce SF’s broader translational relevance in drug delivery and tissue-specific repair.

## 4. Discussion and Outlook

The studies reviewed here show that SF has progressed from a traditional textile fibre to a multifunctional biomaterial platform that can be engineered for structural, therapeutic and diagnostic roles. The adaptability of silk fibroin for biomedical applications rests on three interrelated chemical and structural principles.

First, SF possesses a protein backbone whose secondary structure can be tuned to balance mechanical robustness and biodegradability [10]. As discussed in Section 1, the hierarchical organisation of β-sheet nanocrystals within an amorphous protein matrix governs the stiffness, tensile strength, and degradation kinetics [9]. Modulating the β-sheet content through solvent treatment, annealing, or crosslinking directly alters the crystallinity and therefore the material lifetime, as reflected in hydrogel systems, electrospun membranes, and drug-loaded microspheres, described in Section 2 and Section 3 [56]. This programmable crystallinity distinguishes SF from many synthetic polymers whose degradation pathways are primarily hydrolytic and less structurally coupled.

Second, SF offers a wide aqueous processing window that yields fibres, films, hydrogels, particles, and patterned microstructures [10]. As outlined in Section 2, mild aqueous regeneration after degumming and dissolution enables fabrication across multiple length scales without requiring harsh organic solvents. The same regenerated solution can be electrospun into nanofibres [85], cast into optically transparent films [18,104], processed into injectable hydrogels [47,92], or structured through lithographic techniques [32,33,64]. This versatility underpins the optical fibre sensors, electrospun wound dressings, and controlled-release depots [31,42,43] discussed throughout Section 3.

Third, SF provides a rich functionalization chemistry that enables specific bioactivity without compromising underlying biocompatibility. Reactive side chains (e.g., lysine, tyrosine, aspartic and glutamic acids) allow covalent conjugation via carbodiimide chemistry, enzymatic modification, or blending strategies, as detailed in Section 2.6 [5,48,73]. These chemistries support the incorporation of fluorophores, antibodies, growth factors, nanoparticles, and bioactive peptides while preserving the structural framework of the protein. The nanodiamond–silk hybrids, antibody-functionalized optical fibres, and growth factor-loaded regenerative scaffolds [109] described in Section 3 exemplify this integration of bioactivity with structural stability.

Taken together, these chemically grounded principles explain why SF can function simultaneously as a mechanically competent scaffold and as a bioactive, resorbable interface. Unlike many synthetic polymers that require separate structural and biofunctional components, silk fibroin inherently couples structural programmability with biochemical compatibility [10,56]. In imaging and sensing, SF serves as an optical and chemical stabilisation matrix for a broad range of probes. Nanodiamond–silk hybrids, silk spheres and films loaded with fluorophores, and silk-coated optical fibres demonstrate how SF can reduce aggregation, enhance photon extraction (for high-index emitters such as nanodiamonds), suppress dye leaching and maintain probe activity under physiological conditions. These properties underpin applications from sub-micron imaging probes and theranostic ND–silk spheres to minimally invasive fibre-based pH and protein sensors, wearable all-fibre strain and temperature sensors, and conformal or edible food labels. The common thread is that immobilisation within a silk matrix converts standard optical or electrochemical chemistries into mechanically compliant, biocompatible devices that operate reliably at tissue, skin or food interfaces [31,43,45,74,78]. For tissue engineering and regenerative medicine, SF provides a continuum of structural formats from soft hydrogels to stiff, porous scaffolds and aligned fibrous constructs. Freeze-dried and cryogelated sponges support bone, cartilage and soft-tissue repair, with β-sheet content and crosslinking used to match scaffold degradation to the timescale of tissue remodelling. Electrospun mats and woven SF fibres mimic extracellular matrix architecture and offer contact guidance in skin, nerve, tendon and ligament models, while emerging 3D-printed and bioink-based systems enable spatially patterned, tissue-specific architectures and composite reinforcement. SF-based wound dressings extend these principles to the wound surface: films, foams and nanofibre meshes provide barrier function and moisture balance, while the incorporation of antimicrobials, growth factors or fluorescent reporters adds therapeutic and monitoring capabilities. Across these systems, SF’s benign breakdown products, haemostatic properties and ability to stabilise labile bioactives are as important as its initial mechanical performance [5,56,57].

At the same time, several cross-cutting limitations still hinder translation. The variability in cocoon source and degumming and regeneration protocols leads to differences in molecular weight, β-sheet content and residual sericin, which can alter the mechanics, degradation and immune response; more standardised, reportable processing is needed for reproducible device performance. The long-term stability of immobilised growth factors, enzymes or antibodies under clinically relevant storage and use conditions remains a challenge, as does rigorous toxicology for composite systems that include non-degradable nanomaterials such as nanodiamonds, metal nanoparticles or carbon nanotubes. Finally, many of the most sophisticated SF devices—fibre probes coupled to optical consoles, microneedle patches, or integrated smart textiles—require parallel advances in readout electronics, packaging and regulatory pathways for combination products [13]. From a materials design perspective, it is also important to recognise that the strategies used to “improve” SF properties can introduce new constraints. Blending and cross-linking approaches have been explored to achieve desirable changes in mechanics, thermal stability or water uptake; however, these modifications often come with trade-offs such as increased brittleness, altered degradation profiles or reduced permeability. In addition, some cross-linking chemistries and organic solvent-based processing routes raise concerns about cytotoxicity and residual reagents, requiring careful optimisation and thorough purification before clinical use. These observations emphasise that property enhancement in SF systems is not purely additive and that each modification must be evaluated in the full context of mechanical, biological and regulatory requirements for the intended application [13].

The future development of SF-based systems is likely to build deliberately on its dual role as a structural and functional matrix. In regenerative medicine, hierarchically structured SF composites can couple anisotropic mechanics with the spatially controlled presentation of morphogens and immunomodulatory cues. In sensing and imaging, ultrathin coatings, fibre-based probes, wearable patches and edible labels provide routes for the continuous monitoring of wound status, metabolism, environmental quality and food freshness within a single silk-based platform. In drug delivery, SF particles, films and microneedles support localised, temporally controlled therapy, and when combined with stable optical reporters they enable the non-invasive tracking of carrier fate. If challenges in standardisation, scale-up and long-term safety can be addressed, SF is well positioned to underpin a new generation of integrated biomedical systems that are effective, biodegradable and compatible with both clinical and everyday environments [10,17,52].

## Data Availability

No new data were created or analyzed in this study. Data sharing is not applicable to this article.

## Abbreviations

The following abbreviations are used in this manuscript:

ABTS	2,2′-azino-bis(3-ethylbenzothiazoline-6-sulfonic acid)
BMSC(s)	Bone marrow-derived mesenchymal stem cell(s)
BMP-2	Bone morphogenetic protein-2
COOH	Carboxylic acid groups
ECG	Electrocardiogram
EDC	1-ethyl-3-(3-dimethylaminopropyl)carbodiimide
EMG	Electromyography
FGF-1	Fibroblast growth factor-1
GAG	Glycosaminoglycan
GAGAGS	Glycine–alanine–glycine–alanine–glycine–serine
GOx	Glucose oxidase
H&E	Hematoxylin and eosin
HFIP	Hexafluoroisopropanol
HIF	Hypoxia-inducible factor
HRP	Horseradish peroxidase
IKVAV	Ile-Lys-Val-Ala-Val
IR	Infrared
LC	Inductor capacitor resonant circuit
NHS	N-hydroxysuccinimide
NV	Nitrogen vacancy
ND	Nanodiamond
PLA	Polylactic acid
QD	Quantum dot
RGD	Arginine–glycine–aspartic acid
RSF	Regenerated silk fibroin
SEM	Scanning electron microscopy
SNARF	Seminaphthorhodafluor (pH indicator dye)
SF	Silk fibroin
TGF	Transforming growth factor
VEGF	Vascular endothelial growth factor

## References

[1] Wang H. (2023). Biomaterials in Medical Applications. Polymers.

[2] Wang X., Kluge J.A., Leisk G.G., Kaplan D.L. (2008). Sonication-induced gelation of silk fibroin for cell encapsulation. Biomaterials.

[3] Pritchard E.M., Dennis P.B., Omenetto F., Naik R.R., Kaplan D.L. (2012). Review physical and chemical aspects of stabilization of compounds in silk. Biopolymers.

[4] Reizabal A., Costa C.M., Pérez-Álvarez L., Vilas-Vilela J.L., Lanceros-Méndez S. (2023). Silk Fibroin as Sustainable Advanced Material: Material Properties and Characteristics, Processing, and Applications. Adv. Funct. Mater..

[5] Indrakumar S., Dash T.K., Mishra V., Tandon B., Chatterjee K. (2024). Silk Fibroin and Its Nanocomposites for Wound Care: A Comprehensive Review. ACS Polym. Au.

[6] Cao Y., Wang B. (2009). Biodegradation of silk biomaterials. Int. J. Mol. Sci..

[7] Thurber A.E., Omenetto F.G., Kaplan D.L. (2015). In vivo bioresponses to silk proteins. Biomaterials.

[8] Wang X., Yucel T., Lu Q., Hu X., Kaplan D.L. (2010). Silk nanospheres and microspheres from silk/pva blend films for drug delivery. Biomaterials.

[9] Qi Y., Wang H., Wei K., Yang Y., Zheng R.Y., Kim I.S., Zhang K.Q. (2017). A Review of Structure Construction of Silk Fibroin Biomaterials from Single Structures to Multi-Level Structures. Int. J. Mol. Sci..

[10] Rockwood D.N., Preda R.C., Yücel T., Wang X., Lovett M.L., Kaplan D.L. (2011). Materials fabrication from *Bombyx mori* silk fibroin. Nat. Protoc..

[11] Meyer M. (2019). Processing of collagen based biomaterials and the resulting materials properties. Biomed. Eng. Online.

[12] Johari N., Moroni L., Samadikuchaksaraei A. (2020). Tuning the conformation and mechanical properties of silk fibroin hydrogels. Eur. Polym. J..

[13] Grabska-Zielińska S., Sionkowska A. (2021). How to Improve Physico-Chemical Properties of Silk Fibroin Materials for Biomedical Applications?-Blending and Cross-Linking of Silk Fibroin—A Review. Materials.

[14] Tulachan B., Meena S.K., Rai R.K., Mallick C., Kusurkar T.S., Teotia A.K., Sethy N.K., Bhargava K., Bhattacharya S., Kumar A. (2014). Electricity from the silk cocoon membrane. Sci. Rep..

[15] Zhao M., Qi Z., Tao X., Newkirk C., Hu X., Lu S. (2021). Chemical, Thermal, Time, and Enzymatic Stability of Silk Materials with Silk I Structure. Int. J. Mol. Sci..

[16] Narayanan S., Gokuldas M. (2016). Influence of organic solvents on the structural and thermal characteristics of silk protein from the web of *Orthaga exvinacea* Hampson (Lepidoptera: Pyralidae). J. Chem. Biol..

[17] Omenetto F.G., Kaplan D.L. (2008). A new route for silk. Nat. Photonics.

[18] Khalid A., Lodin R., Domachuk P., Tao H., Moreau J.E., Kaplan D.L., Omenetto F.G., Gibson B.C., Tomljenovic-Hanic S. (2014). Synthesis and characterization of biocompatible nanodiamond-silk hybrid material. Biomed. Opt. Express.

[19] Perotto G., Zhang Y., Naskar D., Patel N., Kaplan D.L., Kundu S.C., Omenetto F.G. (2017). The optical properties of regenerated silk fibroin films obtained from different sources. Appl. Phys. Lett..

[20] Wu R., Ma L., Liu X.Y. (2022). From Mesoscopic Functionalization of Silk Fibroin to Smart Fiber Devices for Textile Electronics and Photonics. Adv. Sci..

[21] Prajzler V., Arif S., Min K., Kim S., Nekvindová P. (2021). All-polymer silk-fibroin optical planar waveguides. Opt. Mater..

[22] Parker S.T., Domachuk P., Amsden J., Bressner J., Lewis J.A., Kaplan D.L., Omenetto F.G. (2009). Biocompatible Silk Printed Optical Waveguides. Adv. Mater..

[23] Zhou Z., Shi Z., Cai X., Zhang S., Corder S.G., Li X., Zhang Y., Zhang G., Chen L., Liu M. (2017). The Use of Functionalized Silk Fibroin Films as a Platform for Optical Diffraction-Based Sensing Applications. Adv. Mater..

[24] Mitropoulos A.N., Perotto G., Kim S., Marelli B., Kaplan D.L., Omenetto F.G. (2014). Synthesis of Silk Fibroin Micro- and Submicron Spheres Using a Co-Flow Capillary Device. Adv. Mater..

[25] Gu J., Li Q., Chen B., Xu C., Zheng H., Zhou Y., Peng Z., Hu Z., Wang B. (2019). Species identification of *Bombyx mori* and *Antheraea pernyi* silk via immunology and proteomics. Sci. Rep..

[26] Minoura N., Aiba S.I., Higuchi M., Gotoh Y., Tsukada M., Imai Y. (1995). Attachment and Growth of Fibroblast Cells on Silk Fibroin. Biochem. Biophys. Res. Commun..

[27] Talukdar S., Mandal M., Hutmacher D.W., Russell P.J., Soekmadji C., Kundu S.C. (2011). Engineered silk fibroin protein 3D matrices for in vitro tumor model. Biomaterials.

[28] Zhang Y., Roohani I. (2025). Recent Advances in Silk Fibroin Derived from *Bombyx mori* for Regenerative Medicine. J. Funct. Biomater..

[29] Huang L., Shi J., Zhou W., Zhang Q. (2023). Advances in Preparation and Properties of Regenerated Silk Fibroin. Int. J. Mol. Sci..

[30] Carriero V.C., Di Muzio L., Petralito S., Casadei M.A., Paolicelli P. (2023). Cryogel Scaffolds for Tissue-Engineering: Advances and Challenges for Effective Bone and Cartilage Regeneration. Gels.

[31] Khalid A., Bai D., Abraham A.N., Jadhav A., Linklater D., Matusica A., Nguyen D., Murdoch B.J., Zakhartchouk N., Dekiwadia C. (2020). Electrospun Nanodiamond-Silk Fibroin Membranes: A Multifunctional Platform for Biosensing and Wound-Healing Applications. ACS Appl. Mater. Interfaces.

[32] Kurland N.E., Dey T., Kundu S.C., Yadavalli V.K. (2013). Precise patterning of silk microstructures using photolithography. Adv. Mater..

[33] Liu W., Zhou Z., Zhang S., Shi Z., Tabarini J., Lee W., Zhang Y., Gilbert Corder S.N., Li X., Dong F. (2017). Precise Protein Photolithography (P3): High Performance Biopatterning Using Silk Fibroin Light Chain as the Resist. Adv. Sci..

[34] Bettinger C.J., Cyr K.M., Matsumoto A., Langer R., Borenstein J.T., Kaplan D.L. (2007). Silk Fibroin Microfluidic Devices. Adv. Mater..

[35] Wenk E., Merkle H.P., Meinel L. (2011). Silk fibroin as a vehicle for drug delivery applications. J. Control. Release.

[36] Numata K., Kaplan D.L. (2010). Silk-based delivery systems of bioactive molecules. Adv. Drug Deliv. Rev..

[37] Lv S. (2020). Silk Fibroin-Based Materials for Catalyst Immobilization. Molecules.

[38] Zhang Y.-Q., Shen W.-D., Xiang R.-L., Zhuge L.-J., Gao W.-J., Wang W.-B. (2007). Formation of silk fibroin nanoparticles in water-miscible organic solvent and their characterization. J. Nanopart. Res..

[39] Giri T.K., Holban A.M., Grumezescu A.M. (2016). 20—Alginate Containing Nanoarchitectonics for Improved Cancer Therapy. Nanoarchitectonics for Smart Delivery and Drug Targeting.

[40] Subia B., Kundu S.C. (2013). Drug loading and release on tumor cells using silk fibroin-albumin nanoparticles as carriers. Nanotechnology.

[41] Khalid A., Mitropoulos A.N., Marelli B., Simpson D.A., Tran P.A., Omenetto F.G., Tomljenovic-Hanic S. (2015). Fluorescent Nanodiamond Silk Fibroin Spheres: Advanced Nanoscale Bioimaging Tool. ACS Biomater. Sci. Eng..

[42] Khalid A., Mitropoulos A.N., Marelli B., Tomljenovic-Hanic S., Omenetto F.G. (2016). Doxorubicin loaded nanodiamond-silk spheres for fluorescence tracking and controlled drug release. Biomed. Opt. Express.

[43] Khalid A., Peng L., Arman A., Warren-Smith S.C., Schartner E.P., Sylvia G.M., Hutchinson M.R., Ebendorff-Heidepriem H., McLaughlin R.A., Gibson B.C. (2020). Silk: A bio-derived coating for optical fiber sensing applications. Sens. Actuators B Chem..

[44] Yucel T., Lovett M.L., Kaplan D.L. (2014). Silk-based biomaterials for sustained drug delivery. J. Control. Release.

[45] Capon P.K., Horsfall A.J., Li J., Schartner E.P., Khalid A., Purdey M.S., McLaughlin R.A., Abell A.D. (2021). Protein detection enabled using functionalised silk-binding peptides on a silk-coated optical fibre. RSC Adv..

[46] Hofmann S., Foo C.T., Rossetti F., Textor M., Vunjak-Novakovic G., Kaplan D.L., Merkle H.P., Meinel L. (2006). Silk fibroin as an organic polymer for controlled drug delivery. J. Control. Release.

[47] Motta A., Migliaresi C., Faccioni F., Torricelli P., Fini M., Giardino R. (2004). Fibroin hydrogels for biomedical applications: Preparation, characterization and in vitro cell culture studies. J. Biomater. Sci. Polym. Ed..

[48] Matthew S.A.L., Seib F.P. (2024). Silk Bioconjugates: From Chemistry and Concept to Application. ACS Biomater. Sci. Eng..

[49] Liu J., Sun H., Peng Y., Chen L., Xu W., Shao R. (2022). Preparation and Characterization of Natural Silk Fibroin Hydrogel for Protein Drug Delivery. Molecules.

[50] Wang L., Li C. (2007). Preparation and physicochemical properties of a novel hydroxyapatite/chitosan–silk fibroin composite. Carbohydr. Polym..

[51] Aigner T.B., DeSimone E., Scheibel T. (2018). Biomedical Applications of Recombinant Silk-Based Materials. Adv. Mater..

[52] Kundu S.C., Reis R.L. (2024). Silk-Based Biomaterials for Tissue Engineering, Regenerative and Precision Medicine.

[53] Freddi G., Anghileri A., Sampaio S., Buchert J., Monti P., Taddei P. (2006). Tyrosinase-catalyzed modification of *Bombyx mori* silk fibroin: Grafting of chitosan under heterogeneous reaction conditions. J. Biotechnol..

[54] Hasturk O., Jordan K.E., Choi J., Kaplan D.L. (2020). Enzymatically crosslinked silk and silk-gelatin hydrogels with tunable gelation kinetics, mechanical properties and bioactivity for cell culture and encapsulation. Biomaterials.

[55] Altman G.H., Horan R.L., Lu H.H., Moreau J., Martin I., Richmond J.C., Kaplan D.L. (2002). Silk matrix for tissue engineered anterior cruciate ligaments. Biomaterials.

[56] Kundu B., Rajkhowa R., Kundu S.C., Wang X. (2013). Silk fibroin biomaterials for tissue regenerations. Adv. Drug Deliv. Rev..

[57] Lovett M., Eng G., Kluge J.A., Cannizzaro C., Vunjak-Novakovic G., Kaplan D.L. (2010). Tubular silk scaffolds for small diameter vascular grafts. Organogenesis.

[58] Oliveira A.L., Sun L., Kim H.J., Hu X., Rice W., Kluge J., Reis R.L., Kaplan D.L. (2012). Aligned silk-based 3-D architectures for contact guidance in tissue engineering. Acta Biomater..

[59] Semmler L., Naghilou A., Millesi F., Wolf S., Mann A., Stadlmayr S., Mero S., Ploszczanski L., Greutter L., Woehrer A. (2023). Silk-in-Silk Nerve Guidance Conduits Enhance Regeneration in a Rat Sciatic Nerve Injury Model. Adv. Healthc. Mater..

[60] Mauney J.R., Nguyen T., Gillen K., Kirker-Head C., Gimble J.M., Kaplan D.L. (2007). Engineering adipose-like tissue in vitro and in vivo utilizing human bone marrow and adipose-derived mesenchymal stem cells with silk fibroin 3D scaffolds. Biomaterials.

[61] Mandal B.B., Grinberg A., Gil E.S., Panilaitis B., Kaplan D.L. (2012). High-strength silk protein scaffolds for bone repair. Proc. Natl. Acad. Sci. USA.

[62] Wang Y., Kim U.-J., Blasioli D.J., Kim H.-J., Kaplan D.L. (2005). In vitro cartilage tissue engineering with 3D porous aqueous-derived silk scaffolds and mesenchymal stem cells. Biomaterials.

[63] Márquez A., Santiago S., dos Santos M.V., Aznar-Cervantes S.D., Domínguez C., Omenetto F.G., Guirado G., Muñoz-Berbel X. (2024). Reusable Colorimetric Biosensors on Sustainable Silk-Based Platforms. ACS Appl. Bio Mater..

[64] Park J., Lee S.-G., Marelli B., Lee M., Kim T., Oh H.-K., Jeon H., Omenetto F.G., Kim S. (2016). Eco-friendly photolithography using water-developable pure silk fibroin. RSC Adv..

[65] Colusso E., Cicerchia L., Rigon M., Gomes V., Martucci A. (2023). Photoluminescence properties of silk–carbon quantum dots composites. J. Sol-Gel Sci. Technol..

[66] Gravier J., Navarro F.P., Delmas T., Mittler F., Couffin A.C., Vinet F., Texier I. (2011). Lipidots: Competitive organic alternative to quantum dots for in vivo fluorescence imaging. J. Biomed. Opt..

[67] Osman A.I., Zhang Y., Farghali M., Rashwan A.K., Eltaweil A.S., Abd El-Monaem E.M., Mohamed I.M.A., Badr M.M., Ihara I., Rooney D.W. (2024). Synthesis of green nanoparticles for energy, biomedical, environmental, agricultural, and food applications: A review. Environ. Chem. Lett..

[68] Capon P.K., Li J., Horsfall A.J., Yagoub S., Schartner E.P., Khalid A., Kirk R.W., Purdey M.S., Dunning K.R., McLaughlin R.A. (2022). A Silk-Based Functionalization Architecture for Single Fiber Imaging and Sensing. Adv. Funct. Mater..

[69] Fu C.-C., Lee H.-Y., Chen K., Lim T.-S., Wu H.-Y., Lin P.-K., Wei P.-K., Tsao P.-H., Chang H.-C., Fann W. (2007). Characterization and application of single fluorescent nanodiamonds as cellular biomarkers. Proc. Natl. Acad. Sci. USA.

[70] Niu L., Shi M., Feng Y., Sun X., Wang Y., Cheng Z., Li M. (2020). The Interactions of Quantum Dot-Labeled Silk Fibroin Micro/Nanoparticles with Cells. Materials.

[71] Tegafaw T., Liu S., Ahmad M.Y., Ali Al Saidi A.K., Zhao D., Liu Y., Yue H., Nam S.-W., Chang Y., Lee G.H. (2023). Production, surface modification, physicochemical properties, biocompatibility, and bioimaging applications of nanodiamonds. RSC Adv..

[72] Benson V., Amini A. (2020). Why nanodiamond carriers manage to overcome drug resistance in cancer. Cancer Drug Resist..

[73] Mane P., Chaudhari R., Qureshi N., Shinde M., Kim T., Amalnerkar D. (2020). Silver Nanoparticles-Silk Fibroin Nanocomposite Based Colorimetric Bio-Interfacial Sensor for On-Site Ultra-Trace Impurity Detection of Mercury Ions. J. Nanosci. Nanotechnol..

[74] Tao H., Brenckle M.A., Yang M., Zhang J., Liu M., Siebert S.M., Averitt R.D., Mannoor M.S., McAlpine M.C., Rogers J.A. (2012). Silk-Based Conformal, Adhesive, Edible Food Sensors. Adv. Mater..

[75] Márquez A., Santos M.V., Guirado G., Moreno A., Aznar-Cervantes S.D., Cenis J.L., Santagneli S.H., Domínguez C., Omenetto F.G., Muñoz-Berbel X. (2021). Nanoporous silk films with capillary action and size-exclusion capacity for sensitive glucose determination in whole blood. Lab. Chip.

[76] Kim D., Cao Y., Mariappan D., Bono M.S., Hart A.J., Marelli B. (2021). A Microneedle Technology for Sampling and Sensing Bacteria in the Food Supply Chain. Adv. Funct. Mater..

[77] Wang J.-Y., Chen L.-J., Zhao X., Yan X.-P. (2023). Silk fibroin-based colorimetric microneedle patch for rapid detection of spoilage in packaged salmon samples. Food Chem..

[78] Wen D.-L., Pang Y.-X., Huang P., Wang Y.-L., Zhang X.-R., Deng H.-T., Zhang X.-S. (2022). Silk Fibroin-Based Wearable All-Fiber Multifunctional Sensor for Smart Clothing. Adv. Fiber Mater..

[79] Meng G., Long F., Zeng Z., Kong L., Zhao B., Yan J., Yang L., Yang Y., Liu X.-Y., Yan Z. (2023). Silk fibroin based wearable electrochemical sensors with biomimetic enzyme-like activity constructed for durable and on-site health monitoring. Biosens. Bioelectron..

[80] Liu X., Zhang W., Lin Z., Meng Z., Shi C., Xu Z., Yang L., Liu X.Y. (2021). Coupling of Silk Fibroin Nanofibrils Enzymatic Membrane with Ultra-Thin PtNPs/Graphene Film to Acquire Long and Stable On-Skin Sweat Glucose and Lactate Sensing. Small Methods.

[81] Liu J., Yang M., Tan J., Yin Y., Yang Y., Wang C. (2022). pH-responsive discoloration silk fibroin films based on prodigiosin from microbial fermentation. Dye. Pigment..

[82] Song Y., Hu C., Wang Z., Wang L. (2024). Silk-based wearable devices for health monitoring and medical treatment. iScience.

[83] Sanjeevappa H.K., Nilogal P., Rayaraddy R., Martis L.J., Osman S.M., Badiadka N., Yallappa S. (2022). Biosynthesized unmodified silver nanoparticles: A colorimetric optical sensor for detection of Hg^2+^ ions in aqueous solution. Results Chem..

[84] González-Restrepo D., Zuluaga-Vélez A., Orozco L.M., Sepúlveda-Arias J.C. (2024). Silk fibroin-based dressings with antibacterial and anti-inflammatory properties. Eur. J. Pharm. Sci..

[85] Aldahish A., Shanmugasundaram N., Vasudevan R., Alqahtani T., Alqahtani S., Mohammad Asiri A., Devanandan P., Thamaraikani T., Vellapandian C., Jayasankar N. (2024). Silk Fibroin Nanofibers: Advancements in Bioactive Dressings through Electrospinning Technology for Diabetic Wound Healing. Pharmaceuticals.

[86] Naik J.T., Rudrakshi C., Prabhuji M.L.V. (2024). A New Horizon in Biocompatible Membrane: The Role of Collagen and Silk Fibroin in Tissue Regeneration-Case series. J. Biol. Med..

[87] He S., Shi D., Han Z., Dong Z., Xie Y., Zhang F., Zeng W., Yi Q. (2019). Heparinized silk fibroin hydrogels loading FGF1 promote the wound healing in rats with full-thickness skin excision. Biomed. Eng. Online.

[88] Su L., Jia Y., Fu L., Guo K., Xie S. (2023). The emerging progress on wound dressings and their application in clinic wound management. Heliyon.

[89] Du P., Chen X., Chen Y., Li J., Lu Y., Li X., Hu K., Chen J., Lv G. (2023). In vivo and in vitro studies of a propolis-enriched silk fibroin-gelatin composite nanofiber wound dressing. Heliyon.

[90] Yang P., Wang D., Shi Y., Li M., Gao M., Yu T., Liu D., Zhang J., Wang J., Zhang X. (2020). Insulin-Containing Wound Dressing Promotes Diabetic Wound Healing Through Stabilizing HIF-1α. Front. Bioeng. Biotechnol..

[91] Shao J., Cui Y., Liang Y., Liu H., Ma B., Ge S. (2021). Unilateral Silver-Loaded Silk Fibroin Difunctional Membranes as Antibacterial Wound Dressings. ACS Omega.

[92] Yan S., Xu S., Wang Y., You J., Guo C., Wu X. (2024). A Hydrogel Dressing Comprised of Silk Fibroin, Ag Nanoparticles, and Reduced Graphene Oxide for NIR Photothermal-Enhanced Antibacterial Efficiency and Skin Regeneration. Adv. Healthc. Mater..

[93] Li S., Renick P., Senkowsky J., Nair A., Tang L. (2021). Diagnostics for Wound Infections. Adv. Wound Care.

[94] Rennie M.Y., Dunham D., Lindvere-Teene L., Raizman R., Hill R., Linden R. (2019). Understanding Real-Time Fluorescence Signals from Bacteria and Wound Tissues Observed with the MolecuLight i:X(TM). Diagnostics.

[95] Safonova L., Bobrova M., Efimov A., Davydova L., Tenchurin T., Bogush V., Agapova O., Agapov I. (2021). Silk Fibroin/Spidroin Electrospun Scaffolds for Full-Thickness Skin Wound Healing in Rats. Pharmaceutics.

[96] Khalid A., Zhang L., Tetienne J.-P., Abraham A.N., Poddar A., Shukla R., Shen W., Tomljenovic-Hanic S. (2019). Intrinsic fluorescence from cellulose nanofibers and nanoparticles at cell friendly wavelengths. APL Photonics.

[97] Tang J., Li X., Wang H., Mai R., Xie M., Tang Y., Huang W., Zhao D., Xiang L. (2025). Application and Prospects of Smart Dressings in Wound Healing. ACS Appl. Mater. Interfaces.

[98] Chou K.C., Chen C.T., Cherng J.H., Li M.C., Wen C.C., Hu S.I., Wang Y.W. (2021). Cutaneous Regeneration Mechanism of β-Sheet Silk Fibroin in a Rat Burn Wound Healing Model. Polymers.

[99] Li Z., Wu N., Cheng J., Sun M., Yang P., Zhao F., Zhang J., Duan X., Fu X., Zhang J. (2020). Biomechanically, structurally and functionally meticulously tailored polycaprolactone/silk fibroin scaffold for meniscus regeneration. Theranostics.

[100] Bhattacharjee P., Kundu B., Naskar D., Kim H.-W., Maiti T.K., Bhattacharya D., Kundu S.C. (2017). Silk scaffolds in bone tissue engineering: An overview. Acta Biomater..

[101] Yang Q., Teng B.H., Wang L.N., Li K., Xu C., Ma X.L., Zhang Y., Kong D.L., Wang L.Y., Zhao Y.H. (2017). Silk fibroin/cartilage extracellular matrix scaffolds with sequential delivery of TGF-β3 for chondrogenic differentiation of adipose-derived stem cells. Int. J. Nanomed..

[102] Sun W., Gregory D.A., Tomeh M.A., Zhao X. (2021). Silk Fibroin as a Functional Biomaterial for Tissue Engineering. Int. J. Mol. Sci..

[103] Kiritani S., Kaneko J., Ito D., Morito M., Ishizawa T., Akamatsu N., Tanaka M., Iida T., Tanaka T., Tanaka R. (2020). Silk fibroin vascular graft: A promising tissue-engineered scaffold material for abdominal venous system replacement. Sci. Rep..

[104] Lawrence B.D., Marchant J.K., Pindrus M.A., Omenetto F.G., Kaplan D.L. (2009). Silk film biomaterials for cornea tissue engineering. Biomaterials.

[105] Shabbirahmed A.M., Sekar R., Gomez L.A., Sekhar M.R., Hiruthyaswamy S.P., Basavegowda N., Somu P. (2023). Recent Developments of Silk-Based Scaffolds for Tissue Engineering and Regenerative Medicine Applications: A Special Focus on the Advancement of 3D Printing. Biomimetics.

[106] Pashutin A., Podbolotova E., Kirsanova L., Dosi O., Efimov A.E., Agapova O., Agapov I. (2025). Silk Fibroin Microparticle/Carboxymethyl Cellulose Composite Gel for Wound Healing Applications. Biomimetics.

[107] Chaala M., Sebba F.Z., Fuster M.G., Moulefera I., Montalbán M.G., Carissimi G., Víllora G. (2023). Accelerated Simple Preparation of Curcumin-Loaded Silk Fibroin/Hyaluronic Acid Hydrogels for Biomedical Applications. Polymers.

[108] Forbes-Jackson L. (2021). The Role of Spider Silk in Peripheral Nerve Regeneration. https://scholarworks.wm.edu/entities/publication/11a28ef4-5173-441e-a8e9-eaff24a065de.

[109] Uebersax L., Mattotti M., Papaloïzos M., Merkle H.P., Gander B., Meinel L. (2007). Silk fibroin matrices for the controlled release of nerve growth factor (NGF). Biomaterials.

[110] Mallepally R.R., Marin M.A., Surampudi V., Subia B., Rao R.R., Kundu S.C., McHugh M.A. (2015). Silk fibroin aerogels: Potential scaffolds for tissue engineering applications. Biomed. Mater..

[111] Dei Rossi G., Buccino F., Longo E., Tromba G., Vergani L.M. (2026). Sustainable silk fibroin scaffolds for bone repair: Assessing their osteogenic potential via AI-enhanced synchrotron imaging workflow. Biomater. Adv..

